# Regulation of PERK–eIF2α signalling by tuberous sclerosis complex-1 controls homoeostasis and survival of myelinating oligodendrocytes

**DOI:** 10.1038/ncomms12185

**Published:** 2016-07-15

**Authors:** Minqing Jiang, Lei Liu, Xuelian He, Haibo Wang, Wensheng Lin, Huimin Wang, Sung O. Yoon, Teresa L. Wood, Q. Richard Lu

**Affiliations:** 1 Key Laboratory of Brain Functional Genomics of STCSM, Institute of Cognitive Neuroscience, East China Normal University, Shanghai 200062, China; 2 Department of Pediatrics, Division of Experimental Hematology and Cancer Biology, Cincinnati Children's Hospital Medical Center, University of Cincinnati, Cincinnati, Ohio 45229, USA; 3 Department of Pediatrics, State Key Laboratory of Biotherapy/Collaborative Innovation Center for Biotherapy, West China Second Hospital, Sichuan University, Chengdu 610041, China; 4 Department of Neuroscience, Institute for Translational Neuroscience, University of Minnesota, Minneapolis, Minnesota 55455, USA; 5 Department of Molecular and Cellular Biochemistry, Center for Molecular Neurobiology, The Ohio State University, Columbus, Ohio 43210, USA; 6 Department of Neurology and Neuroscience, New Jersey Medical School Cancer Center, Rutgers Biomedical and Health Sciences, Newark, New Jersey 07101, USA; 7 Key Laboratory of Birth Defects, Children's Hospital of Fudan University, Shanghai 201102, China

## Abstract

Tuberous sclerosis complex-1 or 2 (TSC1/2) mutations cause white matter abnormalities, including myelin deficits in the CNS; however, underlying mechanisms are not fully understood. TSC1/2 negatively regulate the function of mTOR, which is required for oligodendrocyte differentiation. Here we report that, unexpectedly, constitutive activation of mTOR signalling by *Tsc1* deletion in the oligodendrocyte lineage results in severe myelination defects and oligodendrocyte cell death in mice, despite an initial increase of oligodendrocyte precursors during early development. Expression profiling analysis reveals that *Tsc1* ablation induces prominent endoplasmic reticulum (ER) stress responses by activating a PERK–eIF2α signalling axis and Fas–JNK apoptotic pathways. Enhancement of the phospho-eIF2α adaptation pathway by inhibition of Gadd34-PP1 phosphatase with guanabenz protects oligodendrocytes and partially rescues myelination defects in *Tsc1* mutants. Thus, TSC1-mTOR signalling acts as an important checkpoint for maintaining oligodendrocyte homoeostasis, pointing to a previously uncharacterized ER stress mechanism that contributes to hypomyelination in tuberous sclerosis.

## Abstract (web summary)

The molecular mechanisms regulating myelination are only partially understood. Here authors show that *Tsc1* ablation in oligodendrocyte lineage activates ER stress and apoptotic programs in mice, and that enhancing PERK-eIF2α signalling partially rescues the myelination defects in Tsc1 mutants.

## Graphical abstract

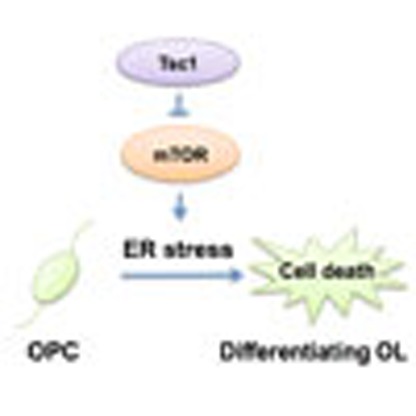

Myelination by oligodendrocytes (OLs) in the mammalian central nervous system (CNS) results in multilayer membrane sheathes that wrap around axons to provide the structural basis for saltatory action potential propagation. Myelinogenesis is a highly complex process that requires cell cycle exit and multi-stage differentiation processes of OL progenitors. Coordination of a complex network of the extrinsic and intrinsic regulatory pathways promotes the maturation of myelinating cells in the CNS^1^,^2^,^3^.

The mammalian-target-of-rapamycin (mTOR), a serine/threonine protein kinase, is a central regulator of the cell growth and necessary for maintenance of metabolic homoeostasis in response to various growth factors, and changes in cellular energy status and amino-acid levels^4^. Tuberous sclerosis complexes 1 and 2 (TSC1 and TSC2; also known as Hamartin and Tuberin) form a heterodimeric protein complex to control mTOR signalling by integrating different signalling pathways: TSC1–TSC2 complex functions as a GTPase-activating protein to inhibit the activity of Rheb, a small GTPase protein that activates mTOR activation^5^,^6^. Mutations in either TSC1 or TSC2 can cause the TCS, a multisystem, autosomal-dominant disorder, which severely affects CNS functions, including cognitive impairment, epilepsy, autism and white matter abnormalities^4^,^7^,^8^,^9^. Genetic TSC loss-of-function mutations are associated with increased phosphorylation of mTOR and its targets, including ribosomal protein S6 (S6) kinase and 4EBP1, and with subsequent increases in protein translation^4^,^10^.

A characteristic feature of TSC in the CNS is white matter aberration, manifested as severe hypomyelination and OL loss^8^,^9^,^11^. The mechanism by which TSC mutations lead to hypomyelination and OL cell death in the TSC disorder remains elusive, and a link between mutant TSC and dysregulation of mTOR has not clearly demonstrated in white matter abnormalities^4^,^7^. In mice, mTOR signalling is required for OL differentiation and CNS myelination^12^,^13^,^14^,^15^. Analyses of mutant mice with ablation of genes-encoding mTOR complex (mTORC) components, Raptor (regulatory-associated protein of mTOR) in mTORC1 and Rictor (rapamycin-insensitive companion of mTOR) in mTORC2, suggest that mTORC1 is the major component in mTOR signalling that regulates CNS myelination in the spinal cord, but not in the brain^13^,^15^. Notably, a balanced TSC-mTOR signalling activity has been suggested to control OL maturation and myelination^13^,^15^.

mTOR signalling regulates protein translational homoeostasis^16^. Recent studies indicate that TSC1/2 loss elevates ER stress or the unfolded protein response, and increases susceptibility to apoptosis^17^,^18^. Myelinating OLs are highly sensitive to ER stress-induced cell death^19^. In response to ER stress, an adaptive program known as the integrated stress response is activated. This stress response can be mediated by the pancreatic ER kinase (PERK), which phosphorylates eukaryotic translation initiation factor 2α (p-eIF2α) to attenuate global protein synthesis and ER stress to maintain proteostasis^20^. This process upregulates transcription factors, such as ATF4, that activate cytoprotective responses and C/EBP-homologous protein (CHOP) expression^19^,^21^, while accumulation of CHOP can induce apoptosis^22^.

Although mTOR signalling activates protein synthesis and is required for myelination, at present, the mechanisms underlying TSC signalling in control of OL myelination are not fully understood. How TSC loss-of-function triggers OL cell death remains unknown. In this report, we demonstrate a critical cell-autonomous role of *Tsc1* for CNS myelination and identify a stage-specific effect of *Tsc1* mutation on OL precursor (OPC) proliferation, survival and differentiation. Strikingly, *Tsc1* mutant mice exhibit extensive OL cell death during the OPC differentiation process. *Tsc1* deletion induces prominent ER stress responses and activation of Fas–JNK-mediated cell death pathways in differentiating OLs. Treatment with guanabenz, which enhances the PERK/p-eIF2α-mediated adaptive response by inhibiting Gadd34-protein phosphatase 1 (PP1), at least in part, rescues OL cell death and myelination defects in *Tsc1*-mutant mice. Taken together, our results demonstrate that TSC-mTOR signalling is a critical for OL homoeostasis and survival programs, and provide a mechanism that might account for OL loss and myelination deficits observed in patients with TSC.

## Results

### *Tsc1* is required for OL differentiation and myelination

**Figure 1:**
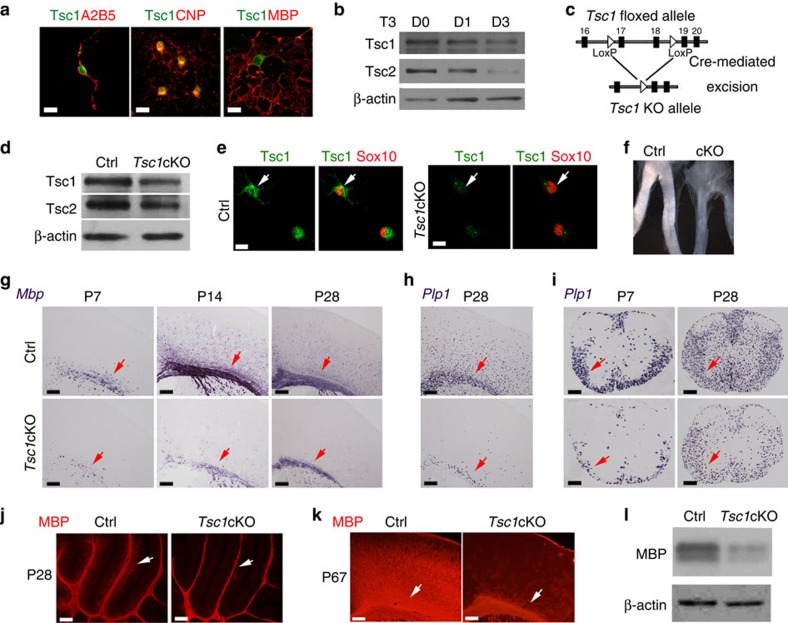
*Tsc1* ablation results in OL differentiation and maturation defects. (**a**) Immunostaining for Tsc1 in OPCs (A2B5^+^), differentiating and maturating OLs (CNP^+^), terminally differentiated OL (MBP^+^) induced by treatment with T3 for 0, 1 and 3 days (left to right), respectively. Scale bar, 10 μm. (**b**) Expression of Tsc1 and Tsc2 assessed by western blot in OPCs treated with T3 for 0, 1 and 3 days. β-actin was used as loading control. (**c**) Schematic diagram of Cre-mediated excision of the floxed *Tsc1* exons 17 and 18, a region of the gene that encodes an essential coiled-coil domain. (**d**) Expression of Tsc1 and Tsc2 in the corpus callosum of control and *Tsc1*cKO mice at P14 was examined by western blot. (**e**) Primary OPCs from control and *Tsc1*cKO mice were immunostained with antibodies to Tsc1 and Sox10. Scale bar, 10 μm. (**f**) The optic nerve of *Tsc1*cKO mice at P12 appears translucent compared with the control. (**g**–**i**) Expression of *MBP* and *Plp1* mRNAs was examined by *in situ* hybridization in the (**g**,**h**) cortex and (**i**) spinal cord of *Tsc1*cKO and control mice at indicated ages. Scale bar, 100 μm. (**j**,**k**) MBP immunostaining in the (**j**) cerebellum and (**k**) cerebral cortex of control, and *Tsc1*cKO at P28 and P67. Scale bar, 100 μm. Arrows indicate white matter tracts. (**l**) Expression of MBP in the corpus callosum of control and *Tsc1*cKO mice at P14 was examined by western blot. β-actin was used as loading control.

Tsc1 expression was detected in the cytoplasm of all of OL lineage cells in culture, including A2B5^+^ OPCs, CNPase^+^ immature OLs and MBP^+^ maturing OLs (Fig. 1a). Western blot analysis indicated that the levels of Tsc1 and Tsc2 decreased gradually as OPCs matured, although both proteins were expressed on all OL lineage cells to some extent (Fig. 1b). To understand the role of Tsc1 in OL development, we generated *Tsc1* knockout (KO) mice by crossing floxed *Tsc1*^fl/fl^ mice with an OL lineage-expressing Olig1-Cre line (Fig. 1c)^23^. The *Tsc1*^fl/fl^:Olig1-Cre^+/−^ mice, referred as *Tsc1*cKO, were born at the normal Mendelian ratio and survived to the adulthood despite the occurrence of sporadic tremors. Reduced Tsc1 levels were confirmed by western blot analysis of the micro-dissected corpus callosum of *Tsc1*cKO mice as compared with controls (*Tsc1*^fl/+^:Olig1-Cre^+/−^) at P14 (Fig. 1d) and by Tsc1 immunostaining of Sox10^+^ OPCs isolated from control and *Tsc1*-mutant brains (Fig. 1e). Expression of the Tsc1 complex partner Tsc2 was also reduced in *Tsc1*cKO mutants (Fig. 1d), consistent with the previously reported role of Tsc1 in stabilizing Tsc2 (ref. 24).

In *Tsc1*cKO mice, the optic nerves from appeared translucent compared with the opaque, white, myelin-enriched control optic nerves (Fig. 1f), indicating a severe CNS myelin defect. The myelin-deficient phenotypes were confirmed by analysing expression of myelin genes such as *Mbp* (myelin basic protein) and *Plp1* (proteolipid protein). Compared with control littermates at P7, P14 and P28, *Mbp* messenger RNA (mRNA) expression was markedly reduced in the *Tsc1*-mutant cortex (Fig. 1g). Similarly, *Plp1* expression was also reduced in the mutant cortex and spinal cord (Fig. 1h,i). Consistently, MBP protein levels were diminished in the cerebellum or cortex of adult *Tsc1* mutants assessed by immunostaining or western blot analysis (Fig. 1j-l), indicating that myelination defects in the CNS of *Tsc1*cKO mice persist into adulthood.

**Figure 2:**
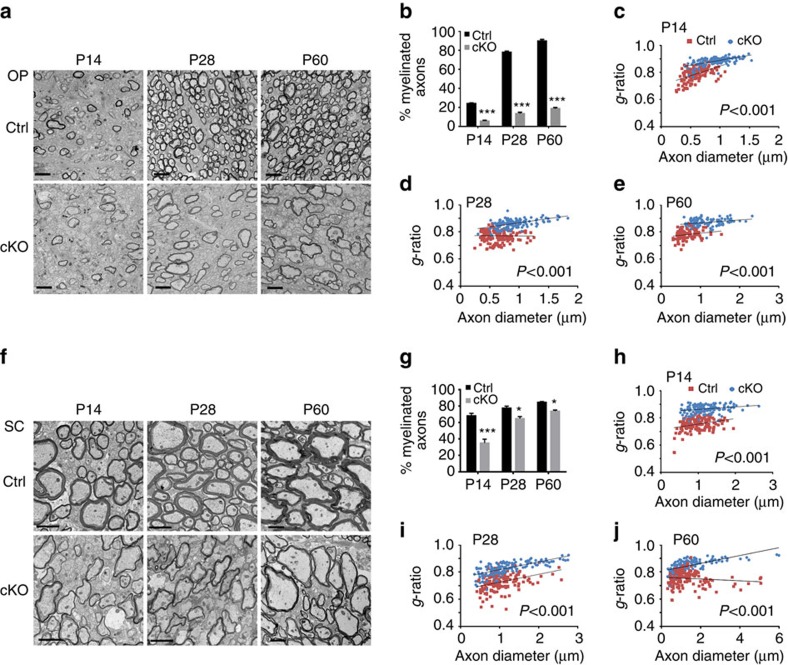
*Tsc1*cKO mice develop myelinogenesis defects. (**a**) Representative electron micrographs of optic nerves in control and *Tsc1*cKO mutants at P14, P28 and P60. Scale bar, 2 μm. (**b**) The percentages of myelinated axons in the optic nerves of control and *Tsc1*cKO mice at indicated stages. Data represent the mean±s.e.m. from three animals; ***P<0.001; Student's *t*-test. (**c**–**e**) *g*-ratio plotted versus axon diameters in the optic nerves of control and *Tsc1cKO* mice at P14 (**c**), P28 (**d**) and P60 (**e**). The data were displayed using scatter plots. *n*=3 animals for each genotype (≥150 myelinating axons were counted for each genotype). Student's *t*-test. (**f**) Representative electron micrographs of the spinal white matter in controls and *Tsc1cKO* mutants at indicated stages. Scale bar, 2 μm. (**g**) The percentages of myelinated axons in the spinal cord white matter of control and *Tsc1*cKO mice at indicated stages. Data represent the mean±s.e.m. from three animals; *P<0.05; ***P<0.001; Student's *t*-test. (**h**–**j**) *g*-ratio plotted versus axon diameters in the spinal white matter of control and *Tsc1cKO* mice at P14 (**h**), P28 (**i**) and P60 (**j**). The data were displayed using scatter plots*. n*=3 animals for each genotype (≥150 myelinating axons were counted for each genotype). Student's *t*-test.

In light of myelin gene downregulation, we further examined myelin sheath assembly in the CNS by electron microscopy. The number of myelinated axons in the optic nerve of *Tsc1*cKO mutants were reduced by ∼70% compared with control mice at P14 and P28, the active myelinogenic stages and in adulthood at P60 (Fig. 2a,b). In the *Tsc1*-mutant optic nerves, myelin *g*-ratio (axon diameter/nerve fibre diameter) was significantly higher than controls at P14, P28 and P60 (Fig. 2c-e), indicating that the thicknesses of myelin sheaths also reduced. Similarly, in the spinal cords of the mutant animals, myelin sheaths were thinner in *Tsc1cKO* mice than controls in all ages tested (Fig. 2f–j). The extent of reduction in the number of myelinated axons was, however, smaller in the spinal cord than in the optic nerve ranging from ∼50% at P14 to ∼13% at P60 (Fig. 2g). Together, these results indicated that *Tsc1* is critical for OL differentiation and proper myelinogenesis in the CNS.

### mTOR hyperactivation contributes to dysmyelinating phenotypes

**Figure 3:**
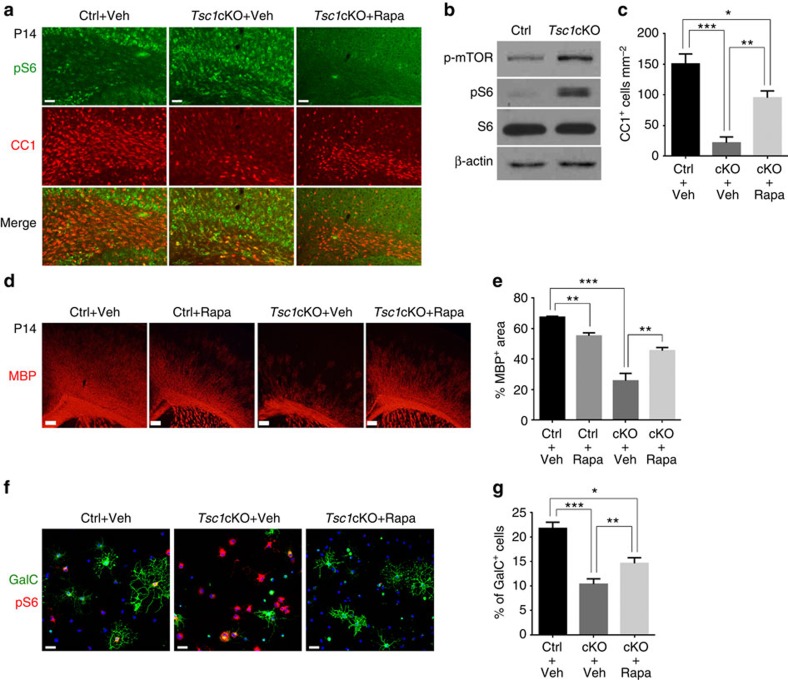
Hyperactivation of mTOR signalling contributes to dysmyelination in *Tsc1* mutants. (**a**) Representative images of the corpus callosum of control and *Tsc1*cKO mutants treated with vehicle and rapamycin from P5 to P14 immunostained with p-S6 and CC1. Scale bar, 50 μm. (**b**) Lysates of control and *Tsc1*cKO corpus callosum at P14 assayed by western blot using antibodies to MBP, p-mTOR, p-S6 and S6. β-actin was used as loading control. (**c**) The number of CC1^+^ cells per mm^2^ from the corpus callosum of control and *Tsc1*cKO mice-treated vehicle and rapamycin. Data represent the mean±s.e.m from four animals per genotype. *P<0.05; **P<0.01; *** *P<*0.001, One-way analysis of variance (ANOVA) with Tukey's multiple-comparison test. (**d**) The corpus callosum of control and *Tsc1*cKO mutants at P14-treated vehicle and rapamycin from P5 to P14 was immunostained with MBP. Scale bar, 100 μm. (**e**) Percentage of MBP area in the cortex of control and *Tsc1*cKO mice treated with vehicle or rapamycin. Data represent the mean±s.e.m from four animals per genotype. *P<0.05; **P<0.01; ***P<0.001. One-way ANOVA with Tukey's multiple-comparison test. (**f**) OPCs isolated from control and *Tsc1*cKO mice were cultured in the differentiation medium for 24 h with vehicle and rapamycin, and immunostained with GalC and p-S6. Scale bar, 50 μm. (**g**) The percentage of GalC^+^ cells in cultures of control and *Tsc1*cKO OPCs. Data represent the mean±s.e.m. from three independent experiments. *P<0.05; **P<0.01; ***P<0.001. One-way ANOVA with Tukey's multiple-comparison test.

Since, Tsc1 is a negative regulator of mTOR activity, we then examined the extent of mTOR activation in *Tsc1*cKO. In the corpus callosum at P14, we observed substantially higher levels of p-S6 in CC1^+^ OLs in *Tsc1*cKO mice than in controls (Fig. 3a). The increase in p-mTOR and p-S6 levels was further confirmed by western blot analysis of corpus callosum extracts (Fig. 3b). These results suggest that the selective deletion of *Tsc1* in OLs resulted in activation of the mTOR pathway. Consistent with this, treatment of *Tsc1*cKO mice with an mTOR inhibitor, rapamycin, from P5 to P14 resulted in a reduction of p-S6 levels (Fig. 3a), and corresponding increases in CC1^+^ OL numbers in the corpus callosum and MBP expression in the cortex compared with vehicle-treated mutant mice (Fig. 3c–e).

It is possible that systemic rapamycin administration influences p-S6 levels in OLs indirectly by inhibiting normal metabolism in neurons. To confirm the cell-autonomous effects of rapamycin on OL differentiation or survival in *Tsc1* mutants, OPCs from the control and *Tsc1*cKO mutants were induced to differentiate in the presence of triiodothyronine (T3). The control OPCs readily differentiated into GalC^+^ maturing OLs after 1 day of T3 treatment, whereas the differentiation of OPCs from *Tsc1*cKO mutants was significantly compromised (Fig. 3f,g). In addition, the number of p-S6^+^ cells increased substantially in *Tsc1*cKO-mutant OLs, and this effect was counteracted by rapamycin (Fig. 3f). Rapamycin also increased the proportion of GalC^+^ OLs in *Tsc1*-mutant OPCs compared with vehicle-treated mutant cells (Fig. 3f,g). These data suggest that hyperactivation of mTOR underlies myelination defects of OLs in *Tsc1* mutants. Notably, the phenotypes we observed with the loss-of-function *Tsc1*, which activates mTOR signalling, are in contrast to published reports wherein selective inactivation of mTOR, Raptor or Rheb1 in the OL lineage led to a delay in myelination or to hypomyelination^12^,^13^,^14^,^15^,^25^.

### Stage-dependent effects of *Tsc1* deletion on OL development

**Figure 4:**
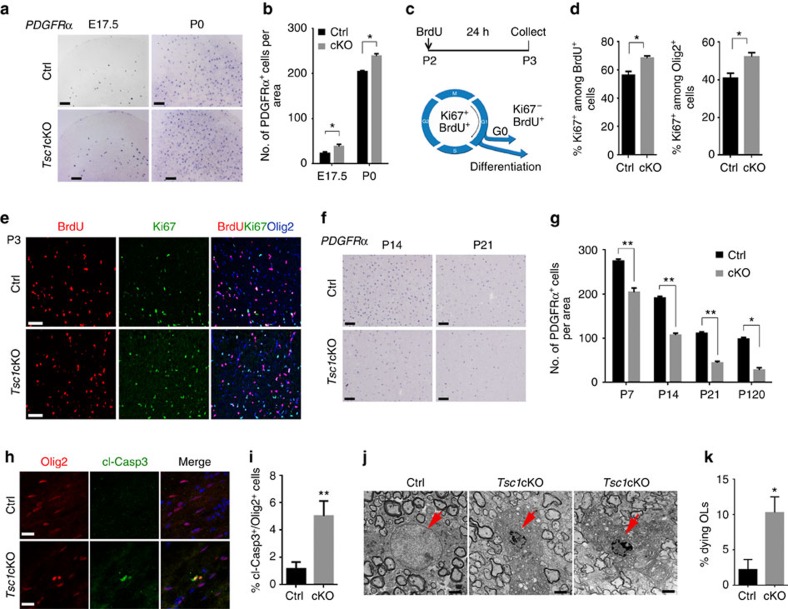
*Tsc1* ablation leads to OPC loss and inhibits cell cycle exit. (**a**) Expression of *PDGFRα* examined by *in situ* hybridization in the cortex of control and *Tsc1*cKO mutants at E17.5 and P0. Scale bar, 100 μm. (**b**) Numbers of PDGFRα^+^ cells in the cortex per unit area (0.4 mm^2^) in control and *Tsc1*cKO mutants at indicated stages. Data represent the mean±s.e.m. from three animals. * *P<*0.05; Student's *t*-test. (**c**) Control and *Tsc1*cKO mice at P2 were pulse-labelled with BrdU for 24 h. Cortical sections collected at P3 were immunostained with antibodies to BrdU, Ki67 and Olig2. (**d**) The percentage of BrdU^+^ and Olig2^+^ OPCs that are Ki67^+^ in control and *Tsc1*cKO cortices. Data represent the mean±s.e.m. from three animals. *P<0.05; Student's *t*-test. (**e**) Cortical sections at P3 were immunostained with BrdU, Ki67 and Olig2. Scale bar, 50 μm. (**f**) Expression of *PDGFRα* by *in situ* hybridization in cortices of control and *Tsc1*cKO mutants at indicated stages. Scale bar, 100 μm. (**g**) Quantification of PDGFRα^+^ OPCs in the cortex per unit area (0.4 mm^2^) in control and *Tsc1*cKO mutants at indicated stages. Data represent the mean±s.e.m. from three animals. **P<0.01; Student's *t*-test. (**h**,**i**) Representative images (**h**) and percentage (**i**) of cells expressing cleaved Caspase 3 (cl-Casp3) among Olig2^+^ cells in the cortex of control and *Tsc1*cKO mutants at P3. Data represent the mean±s.e.m from three animals. **P<0.01; Student's *t*-test. Scale bar, 25 μm. (**j**,**k**) Representative electron microscopy images (**j**) and quantification (**k**) of dying OLs in the spinal cord of *Tsc1*cKO mutants at P14. Data represent the mean±s.e.m from three animals. Scale bar, 2 μm.

In addition to its role in control of OL differentiation, mTOR promotes cell proliferation in a variety of cell types^4^. In agreement with its well-known effect on cell proliferation, a higher number of *PDGFRα*-expressing OPCs were observed at an embryonic stage E17.5 and neonatal stage P0 in *Tsc1*cKO cortices compared with controls (Fig. 4a,b). We then investigated whether the phenotype of increased OPC proliferation was due to a change in cell cycle progression. At P2 (*t*=0 h), mice were treated intraperitoneally with BrdU to label proliferative cells, and the number of cells that were positive for BrdU and for Ki67, a proliferative marker expressed in cycling cells^26^, were analysed after 24 h. In this assay, cells that are labelled with BrdU, but not Ki67 are those that have left the cell cycle (Fig. 4c). We found that the percentage of OPCs that remained in the cell cycle (the percentage of Ki67^+^ cells among BrdU^+^ and Olig2^+^ OPCs) was significantly higher in the *Tsc1*cKO cortex than controls (Fig. 4d,e), suggesting a defect of cell cycle exit in *Tsc1*-mutant OPCs. These data together indicate that *Tsc1* loss enhances OPC proliferation during pre- and neonatal stages by inhibiting their ability to exit the cell cycle, or keeping OPCs at a G2/M phase, consistent with a role of mTOR for regulating G2/M transition^27^. The somas of individual OPCs appeared to be enlarged in the *Tsc1*cKO cortex at P14 relative to control OPCs (Supplementary Fig. 1). To further confirm the proliferation capacity, we performed clonal assay using cultured OPCs. OPCs from *Tsc1* mutants indeed exhibited enhanced proliferation under the cell growth condition compared with controls (Supplementary Fig. 2).

Strikingly, despite the initial increase in OPCs at pre- and neonatal stages in *Tsc1cKO* mice, the number of OPCs was greatly reduced by 40–50% in the cortex at perinatal stages, when OPCs undergo active differentiation processes, and in adulthood compared with controls (Fig. 4f,g). Similar observation was also detected in the developing spinal cord (Supplementary Fig. 3). These data indicate that *Tsc1* loss leads to a modest increase in OPC proliferation during early development, but a substantial reduction of maturing OLs during the myelination phase.

### OL cell death in *Tsc1*-mutant CNS

The reduction OPC and OL numbers during active myelinogenesis in *Tsc1* mutants suggests that OLs might undergo cell death. To test this, we immunostained sections of control and *Tsc1*-mutant brains at P3 for the cleaved form of Caspase 3, a marker for cells undergoing apoptosis^28^. Expression of the cleaved Caspase 3 was detected in Olig2^+^ OLs in the corpus callosum of *Tsc1* mutants, but was hardly detectable in the control (Fig. 4h,i), suggesting that OLs undergo apoptosis during differentiation in *Tsc1* mutants.

Given widespread OL reduction and loss in *Tsc1*cKO mice, we then examined OL morphology in the spinal cord at P14 by electron microscope. In contrast to morphologically normal OLs surrounded by many myelinated axons in control mice, a population of OLs in *Tsc1*cKO mice contained shrunken nuclei, fragmented nuclear envelopes (Fig. 4j,k) or even lost nuclei (Supplementary Fig. 4), characteristic features of cells undergoing apoptosis^29^,^30^. In contrast, we did not detect substantial OL death in age-matched control mice (Fig. 4k). Thus, the observations of OL loss and dying OLs in *Tsc1*cKO mice suggest that sustained mTOR activation causes OLs to undergo apoptotic cell death.

### Tsc1 modulates myelinogenic and ER stress pathways

**Figure 5:**
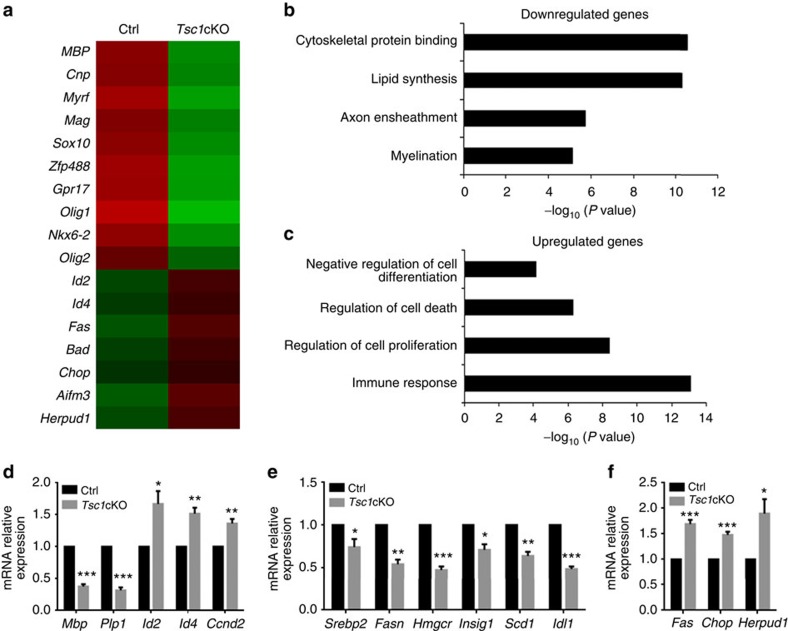
Transcriptome profiling reveals enrichment of the cell survival and apoptotic pathway genes in *Tsc1*cKO mutants. (**a**) Heatmap depicts gene expression of representative myelination-associated and apoptosis-related genes in control and *Tsc1*cKO optic nerves at P12. (**b**,**c**) Gene ontology analyses of pathway enrichment among (**b**) downregulated and (**c**) upregulated genes in *Tsc1*cKO as compared with control. (**d**–**f**) qRT–PCR analysis of representative (**d**) myelination-related genes and differentiation inhibitors, (**e**) lipid synthesis genes, and (**f**) apoptosis and stress response genes from optic nerves of control and *Tsc1*cKO mice at P12. Data represent the mean±s.e.m. from three animals. * *P<*0.05; ** *P<*0.01; *** *P<*0.001; Student's *t*-test.

To investigate whether transcriptional programs were altered in response to *Tsc1* loss of function, we carried out RNA-sequencing (RNA-seq) transcriptome profiling analysis of OL-enriched optic nerves from control and *Tsc1*cKO mice at P12. Myelination-associated genes (for example, *Plp1* and *Mag*) and transcriptional regulators of differentiation (for example, *Nkx6-2*, *Myrf* and *Sox10*) were downregulated, and the expression of differentiation inhibitors such as *Id2* and *Id4* was increased in *Tsc1* mutants relative to controls (Fig. 5a). These data are consistent with OL differentiation defects observed in *Tsc1*cKO mice. Gene ontology analysis indicated that the downregulated genes include those involved in myelination and lipid synthesis (Fig. 5b). In contrast, the upregulated genes are associated with negative regulation of cell differentiation and cell survival, intriguingly, and those involved in inflammation and stress responses (Fig. 5c). Notably, among upregulated genes, we observed substantial enrichment in genes associated with stress responses, including cellular stress sensor *CHOP*, and in genes involved in cell death such as *Fas/CD95*, which encodes a key cell death receptor^31^,^32^, and those encoding pro-apoptotic members of the Bcl-2 family, including *Bad*, *Aifm3* and *Herpud1* (refs 33, 34). Quantitative PCR analyses further confirmed downregulation of *Mbp*, *Plp1*, *Id2*, *Id4* and *Ccnd2*, and upregulation of genes associated with cell stress responses and the apoptotic pathways in *Tsc1*-mutant optic nerves at P12 (Fig. 5d–f). These data suggest that Tsc1-mTOR signalling elevates ER stress response and apoptotic pathways in addition to inhibition of the myelination program.

### Elevation of *Tsc1*-mutant OL cell death upon differentiation

**Figure 6:**
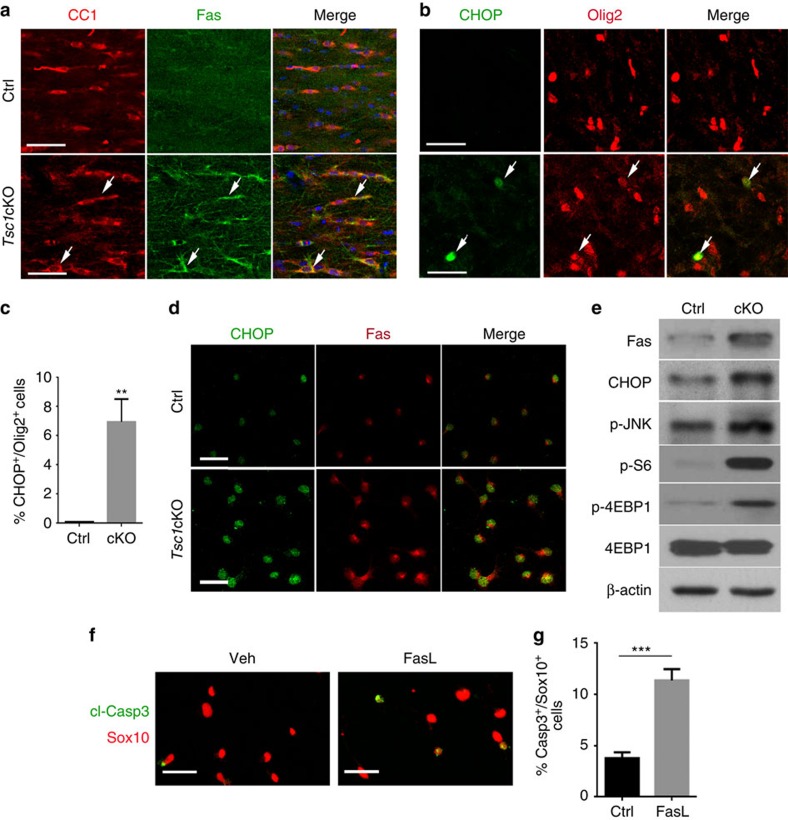
*Tsc1* ablation activates cell apoptotic program. (**a**,**b**) Representative images showing the corpus callosum of P14 control and *Tsc1*cKO mice immunostained with antibodies to (**a**) Fas and CC1, and (**b**) CHOP and Olig2. Scale bars, 25 μm. (**c**) Percentage of CHOP^+^/Olig2^+^ cells in control and *Tsc1*cKO cortices at P14. Data represent the mean±s.e.m. from three animals. *P<0.05; **P<0.01; ***P<0.001; Student's *t*-test. (**d**) Primary OPCs from control and *Tsc1*cKO pups cultured in T3-containing differentiation medium for 24 h were immunostained with CHOP and Fas. Scale bars, 25 μm. (**e**) Western blot analysis of extracts from primary OPCs isolated from control and *Tsc1*cKO animals with antibodies to Fas, CHOP, p-JNK, p-S6 and p-4EBP1; β-actin: loading control. (**f**) Primary OPCs from wild-type mice were treated with solvent and 100 ng ml^−1^ FasL for 24 h, and immunostained with antibodies to cl-Casp3 and Sox10. Scale bar, 25 μm. (**g**) Percentage of cl-Casp3^+^/Sox10^+^ cells from above wild-type OPCs treated with FasL. Data represent the mean±s.e.m. from three independent experiments. ***P<0.001; Student's *t*-test.

To verify the activation of the genes related to ER stress responses and apoptotic signalling, we immunostained control and *Tsc1*-mutant brains with antibodies against Fas and CHOP, which were upregulated at the mRNA level in *Tsc1* mutants. Strikingly, in the corpus callosum at P14, there was a substantial upregulation of Fas expression in CC1^+^ OLs in *Tsc1*cKO mutants (Fig. 6a). In contrast, Fas expression was barely detectable in control mice. Similarly, we detected an increase in expression of CHOP, a key unfolded protein response mediator^32^,^35^,^36^, in a population of Olig2^+^ OL lineage cells in the cortex of *Tsc1*cKO mutants, but not in the control (Fig. 6b,c).

Consistent with the *in vivo* results, primary OPCs isolated from *Tsc1*cKO mice expressed CHOP and Fas at higher levels than control cells under differentiation conditions (Fig. 6d), suggesting a cell-autonomous upregulation of ER stress and apoptotic programs in OLs. Western blot analysis further confirmed the upregulation of Fas, CHOP and phosphorylated JNK (c-Jun N-terminal kinase), known to be involved in cell stress and death pathways^32^,^35^,^36^, along with p-S6 and phosphorylated 4EBP1, the indicators of increased mTOR signalling, during differentiation of *Tsc1*-mutant OPCs compared with controls (Fig. 6e).

Since, Fas protein levels in *Tsc1* mutants were increased both *in vitro* and *in vivo*, we tested whether activation of Fas could induce OL apoptosis by treating wild-type OPCs with FasL, a ligand for Fas. Treatment with FasL significantly increased the percentage of OPCs that underwent cell death (Fig. 6f,g), indicating that the Fas activation triggers apoptotic signalling to cause OL cell death. These results suggest that sustained hyperactivation of mTOR caused by *Tsc1* ablation activates the apoptotic pathway during the onset of OPC differentiation.

### PERK–eIF2α regulates OL survival during differentiation

**Figure 7:**
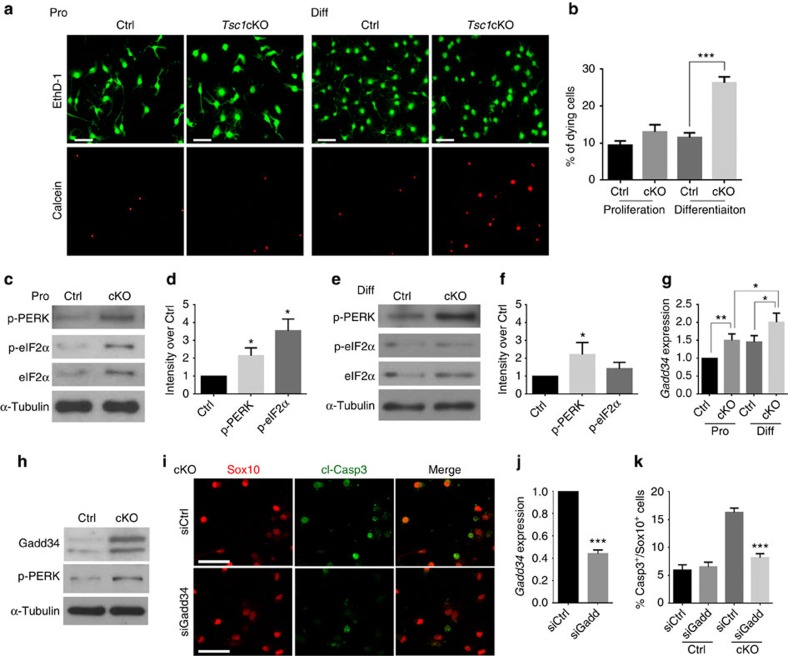
Elevation of adaptive stress responses enhances OL survival. (**a**) OPCs from control and *Tsc1*cKO mutants at P5 were cultured in PDGFAA proliferation (Pro) or T3 differentiation media (Diff) for 24 h and stained with EthD-1 and Calcein AM. Corresponding upper and lower panels were taken in the same field. Scale bar, 50 μm. (**b**) The percentage of dying cells among OPCs isolated from control and *Tsc1*cKO mutants in proliferation or differentiation medium for 24 h. Data represent the mean±s.e.m. from three independent experiments; ***P<0.001, Student's *t*-test. (**c**–**f**) Primary OPCs from control and *Tsc1*cKO pups were cultured under (**c**,**d**) proliferation or (**e**,**f**) differentiation conditions for 24 h. The cell lysates were subject to western blot analysis using antibodies to p-PERK, p-eIF2α and eIF2α; α-tubulin: loading control. p-eIF2α and p-PERK levels were normalized to α-tubulin. The graphs in **d** and **f** depict the fold change of expression levels in *Tsc1*cKO over control cells (data represent the mean±s.e.m. from three independent experiments). *P<0.05; Student's *t*-test. (**g**) qRT–PCR of *Gadd34* from control and *Tsc1*cKO OPCs under proliferation or differentiation conditions for 24 h. Data represent the mean±s.e.m from three independent experiments. *P<0.05; **P<0.01; Student's *t*-test. (**h**) Expression of Gadd34 and p-PERK in the spinal cord of control and *Tsc1*cKO mice at P14 was examined by western blot. α-Tubulin was used as loading control. (**i**) Primary OPCs from control and *Tsc1*cKO mice were treated with Gadd34 siRNA or scramble control siRNA 24 h and immunostained with antibodies to cl-Casp3 and Sox10. Scale bar, 25 μm. (**j**) qRT–PCR analysis of Gadd34 mRNA expression from OPCs transfected with Gadd34 siRNA or scramble control siRNA for 48 h. (**k**) Percentage of cl-Casp3^+^ Sox10^+^ cells from above-treated OPCs in **i**. Data represent the mean±s.e.m from three independent experiments. ***P<0.001; one-way analysis of variance test with Tukey's multiple-comparison test.

Given that the number of OPCs increases during neonatal stages, but decreases during myelinogenesis at the perinatal stage in *Tsc1*-mutant mice, we next sought to determine the state at which oligodendroglia undergo cell death. Under OPC proliferation conditions with platelet-derived growth factor (PDGF)-AA, no significant difference in cell death was detected between *Tsc1*-mutant and control OPCs (Fig. 7a). In contrast, under differentiation-promoting conditions in the presence of T3, we observed a significant increase in cell death among OPCs from *Tsc1* mutants compared with controls, as indicated by labelling with calcein AM, a cell-permeable dye that detects dying cells^37^ (Fig. 7a,b).

To investigate the potential role of the ER stress pathway in survival of *Tsc1*-mutant OLs, we examined ER stress response regulators in OPCs during their differentiation in culture. In proliferating OPCs from *Tsc1*cKO mice, we observed higher levels of p-PERK and p-eIF2α, the ER stress response regulators^19^,^38^, than in OPCs from controls (Fig. 7c,d). eIF2α phosphorylation inhibits synthesis of proteins that adapt cells to the stress^39^. Similarly, expression of the non-phosphorylated form of eIF2α was also increased in *Tsc1*-mutant OPCs (Fig. 7c,d), which is in keeping with upregulation of eIF2α in response to growth stimulation^40^. The *Tsc1*-mutant OPCs, however, underwent cell death upon cell differentiation induced by T3 (Fig. 7a,b). The levels of p-eIF2α and eIF2α were similar in control and *Tsc1*-mutant OPCs in the presence of T3, although the elevation of p-PERK expression persisted in the mutant cells (Fig. 7e,f). Consistently, we detected higher levels of p-eIF2α immunoreactivity in Sox10^+^ OPCs in *Tsc1*-mutant cells than controls under the proliferation, but not differentiation condition in culture (Supplementary Fig. 5). Furthermore, in the corpus callosum of *Tsc1* mutants, we detected that the p-eIF2α signals were upregulated in OPCs, but not mature CC1^+^ OLs compared with controls at P0 and P14 (Supplementary Fig. 6a,b). Accordingly, the p-eIF2a protein level assayed by western blotting was higher at P0 than P14 in the spinal cord of *Tsc1*cKO mice (Supplementary Fig. 6c). These observations raise the possibility that elevation of p-eIF2α-mediated adaptive stress responses may be neuroprotective by preserving OPC viability during early developmental stages.

### Guanabenz treatment protects OLs and enhances myelination

Despite persistent p-PERK upregulation, levels of the PERK effector p-eIF2α were not increased during differentiation of *Tsc1*-mutant OPCs, suggesting that there might be negative feedback control that inhibits eIF2α phosphorylation in differentiating OLs. Previous studies indicated that Gadd34-PP1 can mediate dephosphorylation of p-eIF2α without affecting PERK activity^41^. We therefore evaluated expression of *Gadd34* in *Tsc1*-mutant OPCs under proliferation and differentiation conditions. We found that T3-induced differentiation of *Tsc1*-mutant OPCs resulted in an increase of *Gadd34* expression (Fig. 7g). Consistently, the protein level of Gadd34, along with p-PERK, also increased in the *Tsc1*-mutant spinal cord compared with the control (Fig. 7h). In addition, we found that Gadd34 knockdown by short interfering RNA (siRNA) in *Tsc1*-mutant OPCs reduced cleaved Caspase3-expressing OL under differentiation condition (Fig. 7i–k). These observations suggest that the attenuated p-eIF2α levels might be due to the negative feedback regulation by Gadd34-PP1 during *Tsc1*-mutant OPC differentiation.

**Figure 8:**
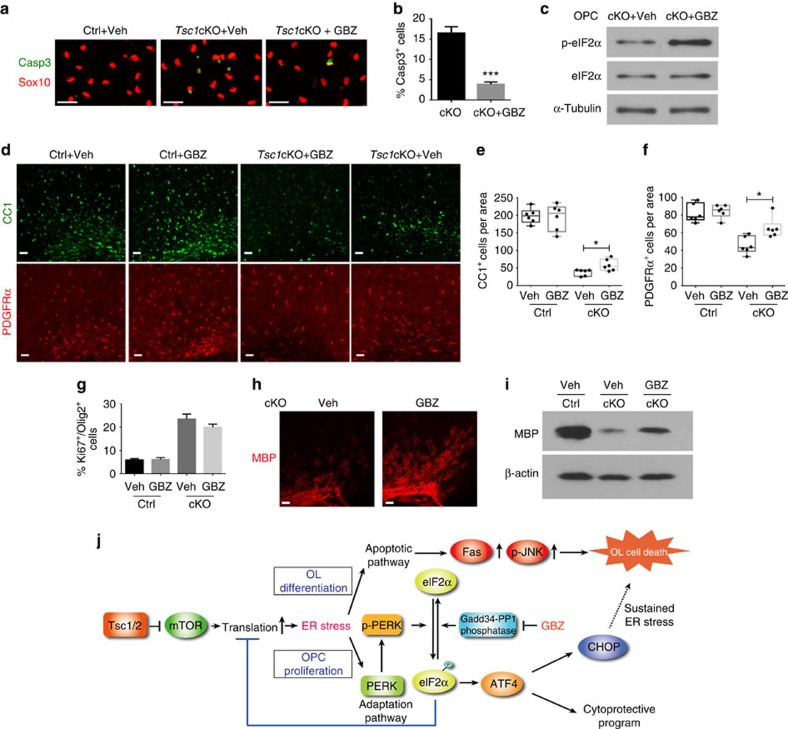
Activation of p-eIF2α-mediated signalling by guanabenz protects OLs and enhances myelination. (**a**) OPCs from control and *Tsc1*cKO mice treated with 5 μM guanabenz for 24 h in T3-containing differentiation medium immunostained with cl-Casp3 and Sox10. Scale bar, 25 μm. (**b**) The percentage of cl-Casp3^+^/Sox10^+^ cells from guanabenz-treated OPCs from control and *Tsc1*cKO mice. Data represent the mean±s.e.m. from three independent experiments. ***P<0.001, Student's *t*-test. (**c**) Western blot analysis of p-eIF2α and eIF2α in OPCs from guanabenz or vehicle-treated *Tsc1*cKO animals; α-tubulin, loading control. (**d**) The corpus callosum of control and *Tsc1*cKO mice treated with vehicle and guanabenz from P7 to P14 was removed at P15, and immunostained with PDGFRα and CC1. Scale bar, 50 μm. (**e**,**f**) Numbers of CC1^+^ cells in the corpus callosum (**e**) and PDGFRα^+^ cells in the cortex (**f**) from vehicle or guanabenz-treated control and *Tsc1*cKO mice. Whiskers in boxplots show the minimum and maximum, boxes extend from the first to the third quartiles with cross lines at the medians. *n*=6 animals per group. *P<0.05. One-way analysis of variance with Tukey's multiple-comparison test. (**g**) Percentage of Ki67^+^ and Olig2^+^ cells from the cortex of control and *Tsc1*cKO mice treated with vehicle or guanabenz. Data represent the mean±s.e.m. from six animals. (**h**) Representative images showing MBP in the corpus callosum of control and *Tsc1*cKO mice treated with vehicle or guanabenz. Scale bar, 50 μm. (**i**) Western blot analysis of MBP in the cortices from control and *Tsc1*cKO animals treated with vehicle or guanabenz; β-actin, loading control. (**j**) A schematic diagram of Tsc1/2 mutation-induced ER stress in OL homoeostasis. *Tsc1* loss leads to mTOR activation, resulting in excessive protein translation and ER stress by activating the PERK–eIF2α–ATF4 adaptation pathway and Fas/p-JNK apoptotic programs. Sustained ER stress coupled with apoptotic pathway activation contributes to the cell death during OL differentiation in *Tsc1* mutants. Inhibition of Gadd34-PP1 by guanabenz (GBZ) enhances p-eIF2α-mediated adaptive responses and partially rescues OL death and myelination defects in *Tsc1* mutants.

Given that phosphorylation of eIF2α is the key event for induction of the adaptive program in response to ER stress, we hypothesize that enhancement of p-eIF2α-mediated signalling protects *Tsc1*-mutant OLs from cell death during differentiation. Guanabenz is known to enhance the level of phosphorylation of eIF2α and adaptive stress response by inhibiting the Gadd34-PP1 phosphatase activity and protein synthesis^42^,^43^. Guanabenz treatment substantially reduced apoptosis in *Tsc1*-mutant OPCs under differentiation conditions compared with vehicle-treated cells (Fig. 8a,b) and protein translation levels, which were elevated in differentiating OLs in *Tsc1* mutants (Supplementary Fig. 7). Increased p-eIF2a was further confirmed by western blotting in guanabenz-treated OPCs (Fig. 8c). Given that guanabenz protected OLs from cell death caused by *Tsc1* deletion *in vitro*, we next investigated whether guanabenz protects OLs in *Tsc1*cKO mutants *in vivo*. Control and *Tsc1*cKO mice were treated daily from P7 until P14 with 8 mg kg^−1^ of guanabenz, a dose that showed neuroprotective effects against INFγ-induced OL death in a previous study^42^. Guanabenz did not have deleterious effects on PDGFRα^+^ OPCs or CC1^+^ OLs in control mice (Fig. 8d–f), or alter the proliferative capacity of Ki67-expressing OPCs in the cortex of both control and *Tsc1* mutants (Fig. 8g). In contrast, guanabenz treatment resulted in a significant increase in the number of CC1^+^ OLs and PDGFRα^+^ OPCs compared with vehicle treatment in *Tsc1*cKO mice (Fig. 8d–f). The extent of myelination assayed by MBP immunostaining and western blot in the cortex was enhanced in guanabenz-treated *Tsc1*-mutant mice (Fig. 8h,i). These *in vitro* and *in vivo* observations indicate that elevation of p-eIF2α by guanabenz treatment protects OLs from cell death and restores myelination at least partially in the absence of Tsc1.

## Discussion

The autosomal-dominant disorder TSC caused by TSC1/2 loss-of-function mutations manifests in neurodevelopmental deficits, including profound hypomyelination and OL loss. Our present findings suggest that the loss of *Tsc1* in OL lineage cells leads to widespread OL death in the CNS by activating the prominent PERK/eIF2α-mediated ER stress and Fas/JNK apoptotic pathways. The sustained ER stresses in *Tsc1* mutants trigger apoptotic programs upon OL differentiation. We find that elevation of p-eIF2α-mediated adaptive responses by guanabenz protects OLs from apoptosis and maintains the balance between stress remediation and cell death in *Tsc1* mutants. Our findings indicate that disruption of OL homoeostasis in *Tsc1* mutants contributes to hypomyelination and OL loss seen in TSC patients, and that modulation of ER stress responses may be beneficial for TSC therapy to restore myelination deficits.

The dysmyelinating phenotype in *Tsc1* mutants is likely mediated through constitutive mTOR activation, since rapamycin partially rescued myelinogenesis defects. Although a hallmark of mTOR signalling activation in organogenesis is to promote cell growth, the present study indicated that sustained mTOR activation in *Tsc1cKO* mice causes detrimental effects on myelinogenesis, consistent with severe myelin defects and OL loss in TSC patients^8^,^9^. We do not observe other CNS abnormalities, including tuber formation and epilepsy in *Tsc1cKO* mice where *Tsc1* is deleted in the OL lineage. Similarly, myelination defect was also detected in *Tsc1* mutants with the floxed *Tsc1* allele deleted by CNP-Cre, in which Cre expression begins in early postmitotic OLs (Supplementary Fig. 8); this is consistent with a recent study with the same Cre line^15^.

Tsc1 and Tsc2 form an integral TSC complex that inhibits mTOR signalling through their GTPase-activating activity on Rheb^5^. We cannot exclude that Tsc2 might have additional unidentified functions that compensate *Tsc1*-mutant phenotypes; however, since the loss of either Tsc1 or Tsc2 abolishes the Rheb-GTPase-activating activity, resulting in constitutively activated mTOR, it is unlikely that Tsc2 alone can suppress mTOR activation in the absence of Tsc1. A recent study suggest that deletion of Tsc2 by Olig2-Cre leads to an extensive gliosis in the corpus callosum, leading to an alteration of cell fate, in addition to a hypomyelination phenotype^44^. In contrast, we do not detect substantial alteration of GFAP expression in the corpus callosum, and the numbers of glutamine synthetase^+^ astrocytes, as well as NeuN^+^ neurons in the cortex of *Tsc1*cKO mice (Supplementary Fig. 9), suggesting that the majority of OPCs, if any, do not adopt an astrocytic fate in *Tsc1*cKO mutants. The phenotypic discrepancy between *Tsc1*cKO and *Tsc2*^fl/fl^;Olig2-Cre mutants might be due to the functional difference in these aspects between Tsc1 and Tsc2, and/or different Cre lines for floxed allele deletion, or other unidentified mechanisms.

The dysmyelination phenotype in *Tsc1* mutants presents an apparent paradox as mTOR is a positive regulator of cell growth and differentiation^5^. mTOR activation promotes protein synthesis through its downstream targets S6 and 4EBP1. Consistent with this, we observed a modest increase of OPC proliferation at early developmental stages in the *Tsc1* mutants, when OPCs undergo proliferation and expansion. Although the trend from increased to decreased OPCs is similar throughout the CNS, the reduction in OPC numbers occurs earlier in the spinal cord than that in the brain (Supplementary Fig. 3), consistent with the fact that the OL differentiation process in the spinal cord precedes that in the brain^45^. The reduction in OPCs may reflect extensive cell death among OLs differentiating from OPCs at the perinatal stages, when the rate of OPC proliferation is not sufficient to replenish the loss of OPC pools. The differentiation process, which induces ER stresses due to enormous demands of myelin protein and lipid biosynthesis, likely contributes to the cell death of differentiating OPCs in *Tsc1* mutants.

There are three branches of ER stress responses that are initiated by distinct transducers located on the ER membrane, including PERK, IRE1-XBP-1 processing^46^,^47^ and ATF6 (ref. 21). We did not detect any substantial alteration of *XBP-1* pre-mRNA splicing or of ATF6 expression levels in *Tsc1* mutants *in vitro* and *in vivo* (Supplementary Fig. 10), suggesting that activation of the p-PERK/p-eIF2α/CHOP signalling axis is the major inducer of ER stress responses in *Tsc1*-deficient OLs.

Balancing of p-eIF2α and eIF2α levels may modulate the cell growth and differentiation processes. Since, phosphorylation of eIF2α leads to shutdown of protein synthesis to mitigate the global cell stress, its upregulation adapts cells to the stress and ensure cell viability^48^. Although the exact mechanisms for p-eIF2α reduction in differentiating OLs is unknown in *Tsc1*cKO mutants, the increase of Gadd34 and its associated phosphatase-promoting activity may at least in part contribute to p-eIF2α reduction. Under proliferation conditions, *Tsc1*cKO OPCs appear to cope with Gadd34 upregulation-induced protein synthesis by enhancing the negative feedback regulator of translation, p-eIF2α and increasing cell proliferation to productively utilize the excess capacity. By contrast, under differentiation conditions, excessive protein synthesis and loads caused by high Gadd34 levels may lead to the increased ER stress levels and reduced p-eIF2α, precluding stress adaption and resulting in OL death. We find that elevation of p-eIF2α levels by guanabenz, which inhibits Gadd34-PP1 phosphatase, enhances the p-eIF2α-mediated adaptive response and restores viability of differentiating OL both *in vitro* and in *Tsc1* mutant animals (Fig. 8). Given that OL maturation requires robust myelin protein production, our observations suggest that *Tsc1*-mutant OPCs under differentiation conditions have impaired adaptive responses and are unable to cope with sustained ER stresses, leading to the cell apoptosis when OPCs are actively undergoing the differentiation process.

We observed an increase in expression of the Fas/CD95 death receptor in *Tsc1*-mutant OLs both *in vitro* and *in vivo*. The Fas receptor belongs to the tumor necrosis factor receptor superfamily and is an important inducer of apoptosis. Fas receptor recruits and activates cysteine protease caspases, particularly procaspases 8 and 10, which in turn activate Caspase 3 and the downstream apoptotic cascades^49^,^50^. In *Tsc1* mutants, the cell death regulation and immune responses were among the most prominently elevated pathways (Fig. 5). The immune response in *Tsc1* mutants might trigger the production of Fas ligands, leading to the activation of Fas signalling and OL death. In addition, recent studies indicate that the elevation of Fas results in trimerization of the receptor, which activates apoptotic cell death independent of its ligands^51^,^52^. Elevated Fas expression is observed in OLs in both chronic active and chronic silent multiple sclerosis demyelinating lesions and contributes to OL cell death^53^,^54^. Since, the activation of Fas signalling has not been reported in other cell types in *Tsc1* mutants, our findings provide an evidence that sustained Tsc1-mTOR signalling results in activation of Fas-mediated apoptosis in addition to ER stress responses during OL differentiation.

Fas signalling transduces cell death signals through the JNK pathway^52^,^55^; we observed upregulation of p-JNK in *Tsc1*-mutant OLs. Thus, constitutive mTOR activation resulting from *Tsc1* loss likely activates the Fas signalling cascade, which further activates JNK-mediated pro-apoptotic signalling (Fig. 8j)^56^,^57^. Although activation of PERK coordinates an adaptive program that promotes cell survival under ER stress^21^,^58^, it may not be solely beneficial since strong PERK activation can be detrimental to OLs through induction of CHOP, a pro-apoptotic factor^33^,^34^. Consistently, CHOP levels are elevated in OLs in vanishing white matter diseases^59^. It should be noted, however, that CHOP has a context-dependent function in OL survival, since CHOP induction enhances survival of ER-stressed OLs in a mouse model of Pelizaeus–Merzbacher disease^60^.

Persistent ER stresses coupled with the Fas/JNK-mediated apoptotic pathway activation likely cause toxicity to OLs; however, other mechanisms may also contribute to the OL cell death in *Tsc1* mutants. It is worth noting that we detected many vacuoles, a hallmark of autophagy, in *Tsc1*-mutant OLs (Supplementary Fig. 4). Since, Tsc1/2 and mTORCs have been shown to regulate autophagy in other cellular contexts^61^,^62^,^63^, it is possible that Tsc1-mTOR signalling may regulate multiple distinct cell death pathways that contribute to OL survival and homoeostasis.

Data from a recent mTOR deletion study indicates that mTOR is not essential for myelination in the brain, but is required for proper myelination in the spinal cord, indicating a region-specific function of mTOR^13^. In the *Tsc1*cKO mutants, we observed hypomyelination throughout the CNS, including the brain and spinal cord, although the defects are more extensive in the optic nerve. This suggests that constitutive activation of mTOR signalling impedes the differentiation of all OL lineage cells, whereas deletion of mTOR mainly impacts spinal cord myelination.

Our findings of differential effects in OPC proliferation and differentiation following *Tsc1* deletion indicate that Tsc1-mTOR signalling has biphasic, context-dependent effects on cell proliferation and apoptosis. This is particularly interesting as mTOR signalling activation is required for myelination in the CNS^12^,^13^,^15^,^64^. Thus, a balance of mTOR activity is required to ensure the expansion of OPC pools during the early phase of development, and their subsequent differentiation and myelination at postnatal stages^15^. In *Tsc1* mutants, a population of OLs survives and produces thinner myelin. It is possible that the extent of ER stress may vary among individual OLs. Moreover, environmental cues surrounding individual OLs are different, and may impact OL viability and myelination capacity. These factors may contribute to whether an OL undergo cell death or survive, but with a defect in myelinogenesis.

TSC1/2 are important sensors of a variety of environmental factors, including amino acids, growth factors, cytokines and hormones, such as insulin and insulin-like growth factor 1, which are important for OL development^27^, and regulation of cell growth and metabolism^4^. How TSC1/2 integrate these signals to coordinate OPC proliferation and differentiation during the myelination process remains to be elucidated. Our data further indicate that constitutive activation of mTOR signalling may not be a viable therapeutic option to promote myelination in the CNS. Nonetheless, since *Tsc1* inactivation enables initial OPC proliferation during early development, OPC production and subsequent OL regeneration might be enhanced by transient *Tsc1* expression blockade or mTOR activation confined to a therapeutic window. Furthermore, by exploring the cell growth pathways such as through TSC-mTOR and PI3K-AKT signalling^65^, as well as the integrated stress response signalling that controls cell survival and death, it may be possible to design a strategy to promote survival of OLs and myelin repair in patients with TSC or, perhaps, the devastating central demyelinating diseases such as multiple sclerosis.

## Methods

### Animals

Tsc1^fl/fl^ mice (Tsc1<tm1Djk>/J – 005680; The Jackson Laboratory) were crossed with Olig1-Cre^23^ or CNP-Cre^66^ hemizygous mice to produce control (Tsc1^fl/+^;Cre^+/−^) and *Tsc1*cKO offspring. Animals of either sex were used in the study and littermates were used as controls unless otherwise indicated. The mouse strains used in this study were generated and maintained on a mixed C57Bl/6;129 Sv background and housed in a vivarium with a 12-h light/dark cycle. All animal use and studies were approved by the Institutional Animal Care and Use Committee of the Cincinnati Children's Hospital Medical Center.

### Tissue and histology

The brain, spinal cord and optic nerves of mice at defined ages were dissected and fixed overnight in 4% paraformaldehyde, and processed for vibratome- or cryo-sectioning. RNA *in situ* hybridization was performed using digoxigenin-labelled riboprobes (murine *PDGFRα, Plp1/Dm-20* and *Mbp*)^45^.

### Electron microscopy

Tissue processing was performed essentially as described previously^67^. In brief, mice were deeply anaesthetised with ketamine/xylazine, perfused with 0.1 M cacodylate, followed by 4% paraformaldehyde/2.5% glutaraldehyde in 0.1 M cacodylate (pH 7.2). Spinal cord and optic nerves were dissected, post fixed in 1% OsO4, dehydrated through a graded ethanol series, infiltrated in propylene oxide and embedded in resin. Semithin sections were stained with toluidine blue and ultrathin sections were stained with lead citrate.

### Immunohistochemistry and immunoblotting

Cryosections or vibratome sections were permeabilized and blocked in blocking buffer (0.3% Triton X-100 and 5% normal donkey serum in phosphate-buffered saline (PBS)) for 1 h at room temperature and overlaid with primary antibodies overnight at 4 °C. Antibodies used in the study were: rabbit anti-Olig2 (Millipore, AB9610, 1:2,000), rat anti-PDGFRα (BD Bioscience, 558774, 1:500), mouse anti-APC (CC1, Oncogene Research, OP80, 1:500), goat anti-MBP (Santa Cruz Biotechnology, sc-13914, 1:500), Tsc1 (Pierce PA5-18506, 1:200), Fas (Santa Cruz sc-1024, 1:200), CHOP (Pierce MA1-250, 1:200), p-PERK (Cell Signaling 3179, 1:2,000), ATF6 (Abcam ab122897, 1:2,000), p-mTOR (Cell Signaling 2974, 1:2,000), P-S6 (Cell Signaling 2211, 1:2,000), S6 (Cell Signaling 2217, 1:2000), p-eIF2α (Cell Signaling 9721, 1:1,000), eIF2α (Cell Signaling 9722, 1:2,000), p-4EBP1 (Cell Signaling 9451, 1:2,000), Gadd34 (Santa Cruz sc-8327, 1:200) and p-JNK (Cell Signaling 9251, 1:1,000), NG2 (Millipore MAB5384, 1:1000), BrdU (Abcam ab6326, 1:500) and Ki67 (Thermo Fisher RM9106-S0, 1:500). After washing in PBS, cells or sections were incubated with secondary antibodies conjugated to Cy2, Cy3 or Cy5 (Jackson ImmunoResearch Laboratories, 1:1,000) for 2 h at room temperature, stained in DAPI for 10 min, washed in PBS and mounted with fluoromount-G (SouthernBiotech). The fluorescence images in the corresponding CNS regions between control and mutants were acquired under a Nikon E-C2 confocal microscope system, and quantified in a double-blinded manner by ImageJ. Images from at least five sections per animal were collected for analysis. For immunoblotting, white matter, spinal cord and whole-cell lysates using 1 × passive lysis buffer (Promega, Madison Cat# E194) supplemented with a protease inhibitor cocktail (1:200, Sigma, St Louis, Missouri, USA). After western blotting, proteins were detected with appropriate secondary antibodies by using chemiluminescence with the enhanced chemiluminescence (ECL) kit (Pierce) according to manufacturer's instructions. Western blot Images have been cropped for presentation. Images have been cropped for presentation. Full-size images for the main figures are presented in Supplementary Figs 11 and 12 and for the Supplementary Figs are in Supplementary Fig. 13, respectively.

### Primary oligodendroglial cell culture and transfection

Primary rat OPCs were isolated from cortices of pups at P2 using a differential detachment procedure^68^. After removing microglia and astrocytes through shaking the mixed glia-culture and differential attachment, isolated rat OPCs were grown in the OPC growth medium (Sato medium supplemented with mitogens 10 ng ml^−1^ PDGFAA and 20 ng ml^−1^ basic fibroblast growth factor), and differentiated in the OL differentiation medium (Sato medium supplemented with 15 nM T3 and 10 ng ml^−1^ ciliary neurotrophic factor). Mouse OPCs were isolated from neonatal or perinatal cortices of control and mutants by immunopanning with antibodies Ran-2, GalC and O4 or PDGFRα sequentially^69^,^70^. The isolated mouse OPCs were cultured in the OPC growth medium plus B27, 1 ng ml^−1^ NT3 and 5 μM forskolin.

siRNA transfection in primary OPCs were carried out by lipofectamine RNAiMAX (Invitrogen Inc.). siRNAs were purchased from Sigma-Aldrich with the following catalogue numbers: control siRNA: MISSION siRNA Universal Negative Control #1 SIC001; and Gadd34 siRNA, SASI_Mm01_00192455. In the FasL-induced apoptosis assay, OPCs were incubated with 100 ng ml^−1^ FasL (ALX-522-001-C010; Enzo Life Sciences, Inc.) without PDGFAA for 24 h. For OPC clonal proliferation assay, 1,000 cells ml^−1^ per well were plated at a clonal density in 12-well plate in the growth medium. Single cells were marked and observed under microscope everyday.

In the protein translation assay, isolated OPCs from control and *Tsc1*cKO mutants were cultured with T3 for 24 h with solvent or guanabenz. Newly synthesized proteins were then assessed by Click-iT HPG Alexa Fluor 488 Protein Synthesis Assay kit (Thermo Fisher Scientific C10428) according to manufacturer' instructions. Click-iT HPG (L-homopropargylglycine) is an amino-acid analogue of methionine for monitoring protein translation.

### Rapamycin and guanabenz treatment

Rapamycin powder (Sirolimus; LC Laboratories) was dissolved in ethanol and stored at a stock concentration of 25 mg ml^−1^ in aliquots at −20 °C. Working solution was prepared freshly before use with a final concentration of 1 mg ml^−1^ rapamycin in 4% ethanol, 5% Tween 80 and 5% PEG400. Mice were administered daily intraperitoneal injections with either rapamycin (10 mg kg^−1^ body weight) or vehicle once per day from P5 to P14. Mice were then collected and analysed by immunohistochemistry. For primary mouse OPC treatment, 15 ng μl^−1^ rapamycin working solution was mixed in the differentiation medium.

Guanabenz (G110, Sigma) was dissolved in water at 2 mg ml^−1^. Mice were administered daily intraperitoneal injections with either guanabenz (8 mg kg^−1^ body weight) or vehicle once per day from P7 to P14 (*n*=6 each group per genotype). At P15, the brains of vehicle- or guanabenz-treated mice were collected and analysed by immunohistochemistry. Images of corresponding brain regions were quantified in a blinded manner. Sampling size of mice in each treatment group was determined based at least 80% power for detecting a difference using a two group *t*-test with a 0.05 two-sided significance level. Animal groups were randomized during the treatment. We used six animals per group per genotype to achieve at least 80% power to detect the difference with 95% confidence.

### RNA extraction and qRT–PCR

Total RNA was extracted per the Trizol (Life Technologies) protocol. Complementary DNA was generated with iScript cDNA Synthesis kit (Bio-Rad). Quantitative PCR with reverse transcription (qRT–PCR) was performed using the ABI Prism 7700 Sequence Detector System (Perkin-Elmer Applied Biosystems). qRT–PCR primers for mouse gene sequences were: Plp1-f, tgctcggctgtacctgtgtacatt and Plp1-r, tacattctggcatcagcgcagaga; Mbp-f, tcacagaagagaccctcaca; Mbp-r, gccgtagtgggtagttcttg; Fas-f, cttgctggctcacagttaaga and Fas-r, gggcctccttgatataatccttc; Herpud1-f, ggaggtatggacccagaaatg and Herpud1-r, ctggaagaagagaggcaaagaa; CHOP-f, ggaagagcaaggaagaactagg and CHOP-r, agctagctgtgccactttc; Ccnd2-f, ctcccgcagtgttcctattt and Ccnd2-r, tcacagacctctagcatcca; Gadd34-f, gacccctccaactctccttc and Gadd34-r, cttcctcagcctcagcattc; and Gapdh-f, tgccaaatatgatgacatcaagaa and Gapdh-r, ggagtgggtgtcgctgttg. XBP1 splicing primers are: 410-S, acacgcttgggaatggacac; and 580-A, ccatgggaagatgttctggg.

### RNA-seq and data analysis

We isolated RNAs from the optic nerves of control and mutant mice at P12, and subjected samples to RNA-seq. RNA-seq libraries were prepared using Illumina RNA-seq Preparation kit (Illumina) and sequenced on a HiSeq 2000 sequencer. RNA integrity numbers for RNA quality assessment were larger than nine by Agilent RNA 6000 Nano kit (Agilent 2100 Bioanalyzer system, Agilent Technologies, Inc.). The depth of sequencing coverage was ∼20 million reads per sample. RNA-seq reads were mapped using TopHat with default settings (http://tophat.cbcb.umd.edu). TopHat output data were then analysed by Cufflinks to (1) calculate fragments per kilobase of transcript per million mapped reads (FPKM) values for known transcripts in mouse genome reference, and (2) test the changes of gene expression between *Tsc1*cKO and control. Heatmap of gene differential expression was generated using Gene cluster and Java Treeview. Gene ontology functional classifications were performed using DAVID (http://david.abcc.ncifcrf.gov) and Ingenuity Pathway analysis.

### Statistic analysis

All data analyses were done using GraphPad Prism 6.00 (San Diego, California, USA www.graphpad.com). Data are shown as mean±s.e.m. or as a box-and-whisker plot. The animals with the same genotypes at the same age exhibit very similar phenotypes. We have also used littermates as controls to minimize the background difference. Quantifications were performed from at least three independent experiments and quantified blindly. Data distribution was assumed to be normal, but this was not formally tested. For cell-based and gene expression assays, as well as animal phenotype analysis, no statistical methods were used to predetermine sample sizes, but our sample sizes are similar to those generally employed in the field. For drug treatment study in mice, animal groups were randomized during treatment, and at least four animals per group per genotype were used to achieve adequate power (>80%) to detect the difference with 95% confidence. Statistical significance is determined using unpaired Student's *t*-test between two groups. One-way analysis of variance test was performed with multiple comparisons or pairwise comparisons following Tukey's ranking tests when comparing multiple groups. *P*<0.05 is considered to be statistically significant.

### Data availability

The data that support the findings of this study are available from the corresponding author upon reasonable request.

## Additional information

**Accession codes:** All the RNA-seq data have been deposited in the NCBI Gene Expression Omnibus (GEO) under accession number GSE47893.

**How to cite this article:** Jiang, M. *et al*. Regulation of PERK–eIF2α signalling by tuberous sclerosis complex-1 controls homoeostasis and survival of myelinating oligodendrocytes. *Nat. Commun.* 7:12185 doi: 10.1038/ncomms12185 (2016).

## Supplementary Material

Supplementary Information Supplementary Figures 1-13
