# Corrigendum: Regulation of PERK–eIF2α signalling by tuberous sclerosis complex-1 controls homoeostasis and survival of myelinating oligodendrocytes

**DOI:** 10.1038/ncomms13767

**Published:** 2016-11-22

**Authors:** Minqing Jiang, Lei Liu, Xuelian He, Haibo Wang, Wensheng Lin, Huimin Wang, Sung O Yoon, Teresa L Wood, Q. Richard Lu

Nature Communications 7: Article number: 12185; DOI: 10.1038/ncomms12185 (2016); Published: 07 15 2015; Updated: 11 22 2016

The GEO accession number provided in this article is incorrect; the correct number is GSE74893.

