# Supplementary material for: Regulation of PERK–eIF2α signalling by tuberous sclerosis complex-1 controls homoeostasis and survival of myelinating oligodendrocytes
Source: Nat Commun. 2016 Jul 15;7:12185. doi: 10.1038/ncomms12185 (PMC4947172; doi:10.1038/ncomms12185)
Supplement: Supplementary Information — Supplementary Figures 1-13 [file ncomms12185-s1.pdf]

SUPPLEMENTARY INFORMATION
Jiang et al.

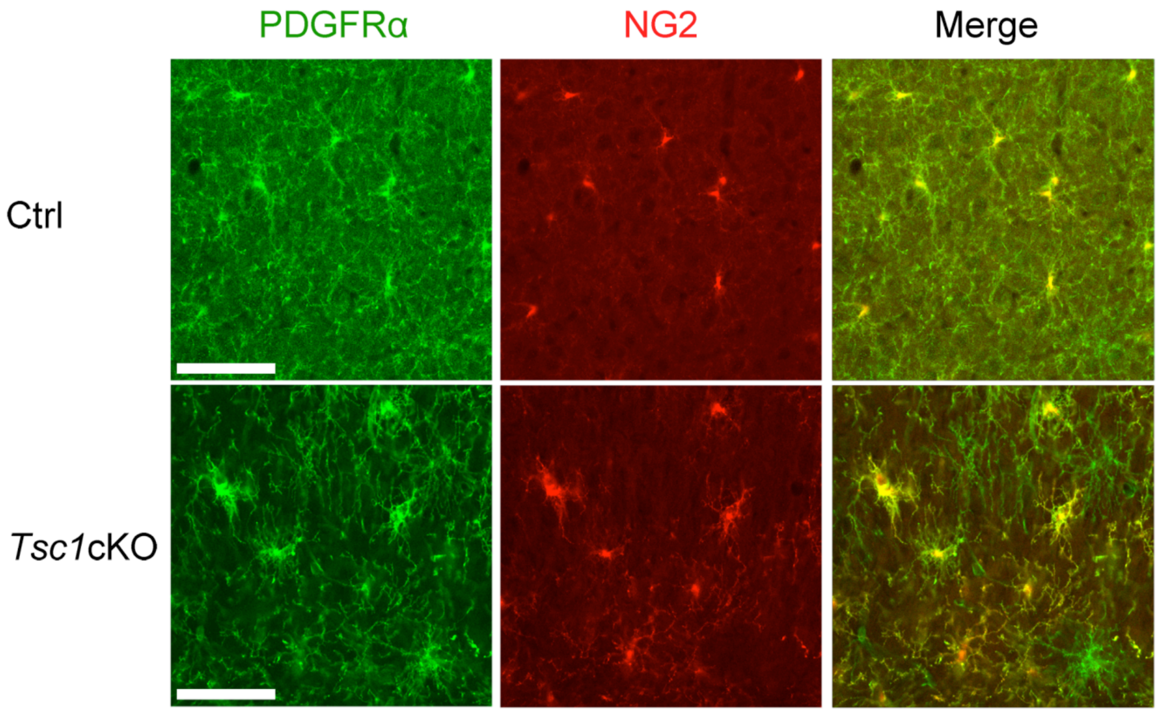

Supplementary Figure 1. Enlarged NG2$^+$ and PDGFRα$^+$ cells in the *Tsc1*cKO cortex

Immunofluorescent signals of PDGFRα (green) and NG2 (red) in the cortex of control and *Tsc1*cKO mutant at P14. Scale bars, 50 µm.

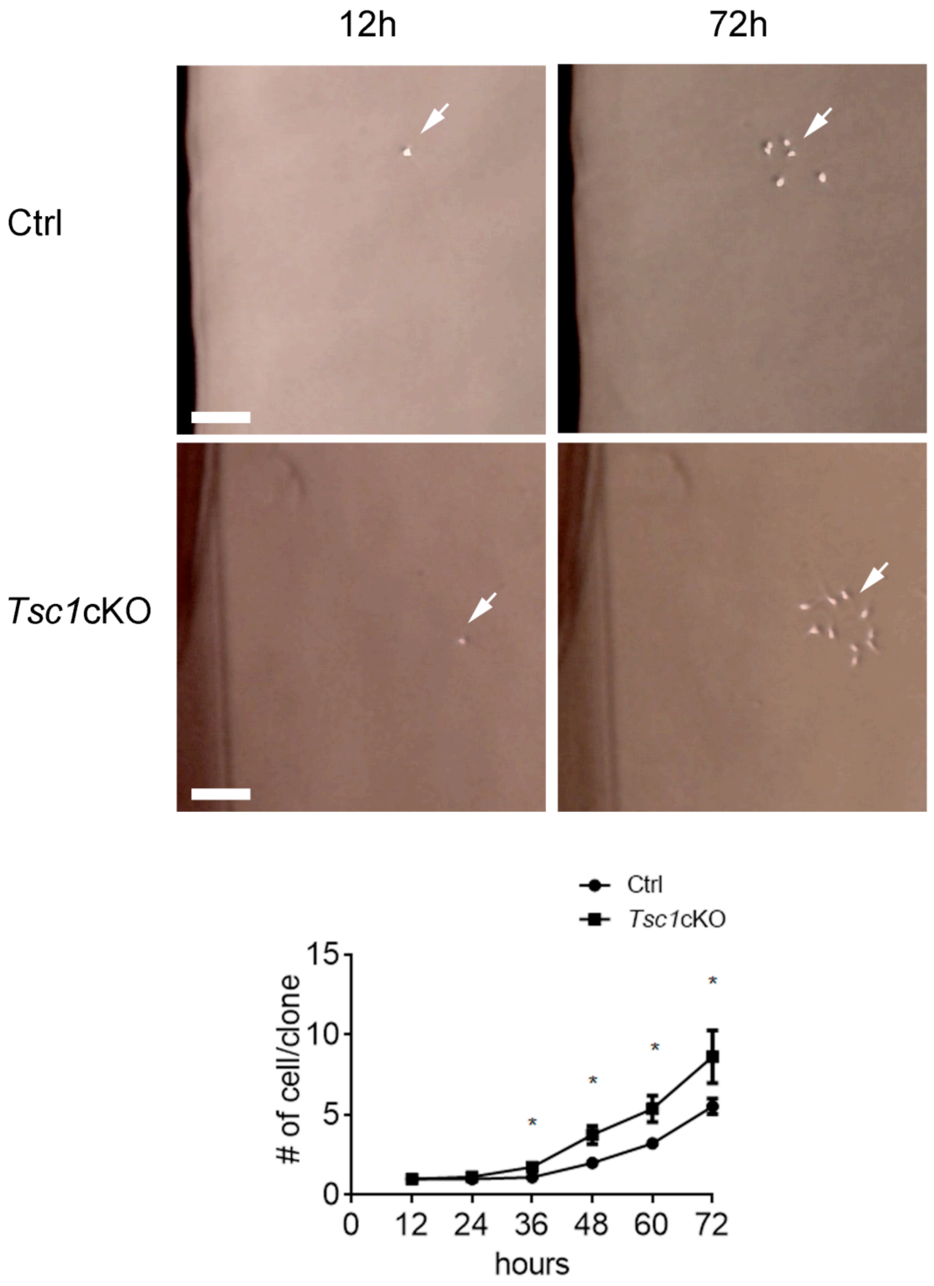

Supplementary Figure 2. *Tsc1*-mutant OPCs exhibit enhanced cell growth

(**a**) OPCs isolated from the perinatal stage at P10 were cultured at the clonal density (1000 cells/ml/well) in the 12-well plate in the mitogen-containing growth medium. Representative images showing the growth of individual OPC from control and *Tsc1*cKO mutants at 12 and 72 h in culture.
(**b**) The number of control and *Tsc1*cKO OPCs under the growth condition over the course of time in the clonal assay. Data represent the mean ± s.e.m. from 12 single cells/genotype. Scale bars, 50μm. * $P < 0.05$, Student's *t* test

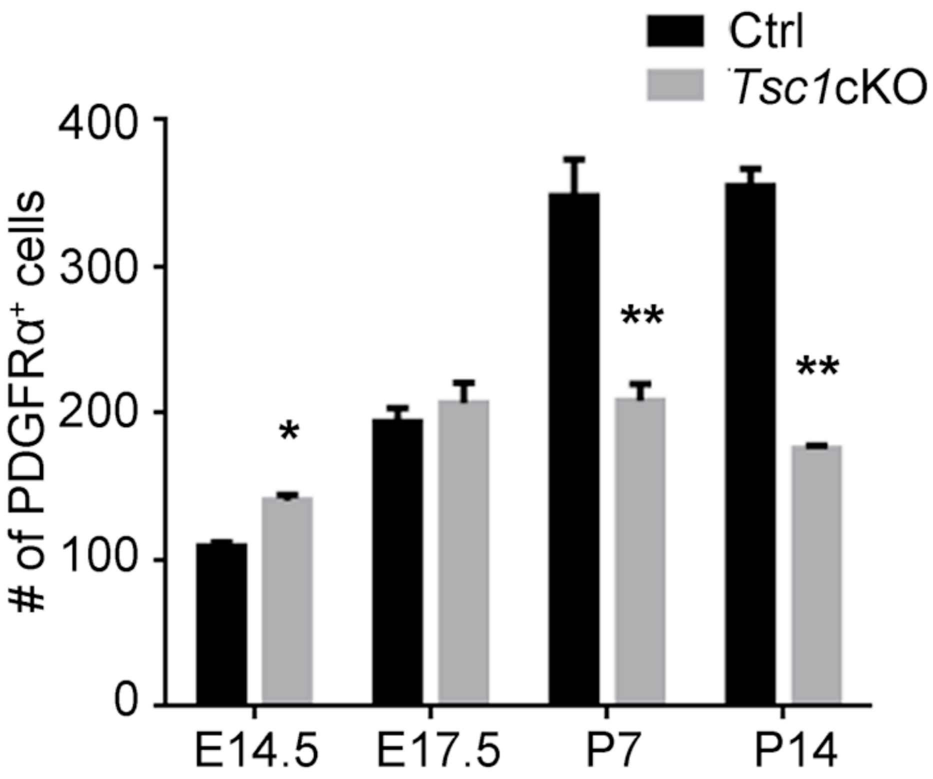

Supplementary Figure 3. OPC development in the *Tsc1*cKO spinal cord

Numbers of *PDGFRα*$^{+}$ cells in the spinal cord at the thoracic level in control and *Tsc1*cKO mutants at indicated stages by in situ hybridization. Data represent the mean ± s.e.m. from three animals. * $P < 0.05$, ** $P < 0.01$, Student's *t* test.

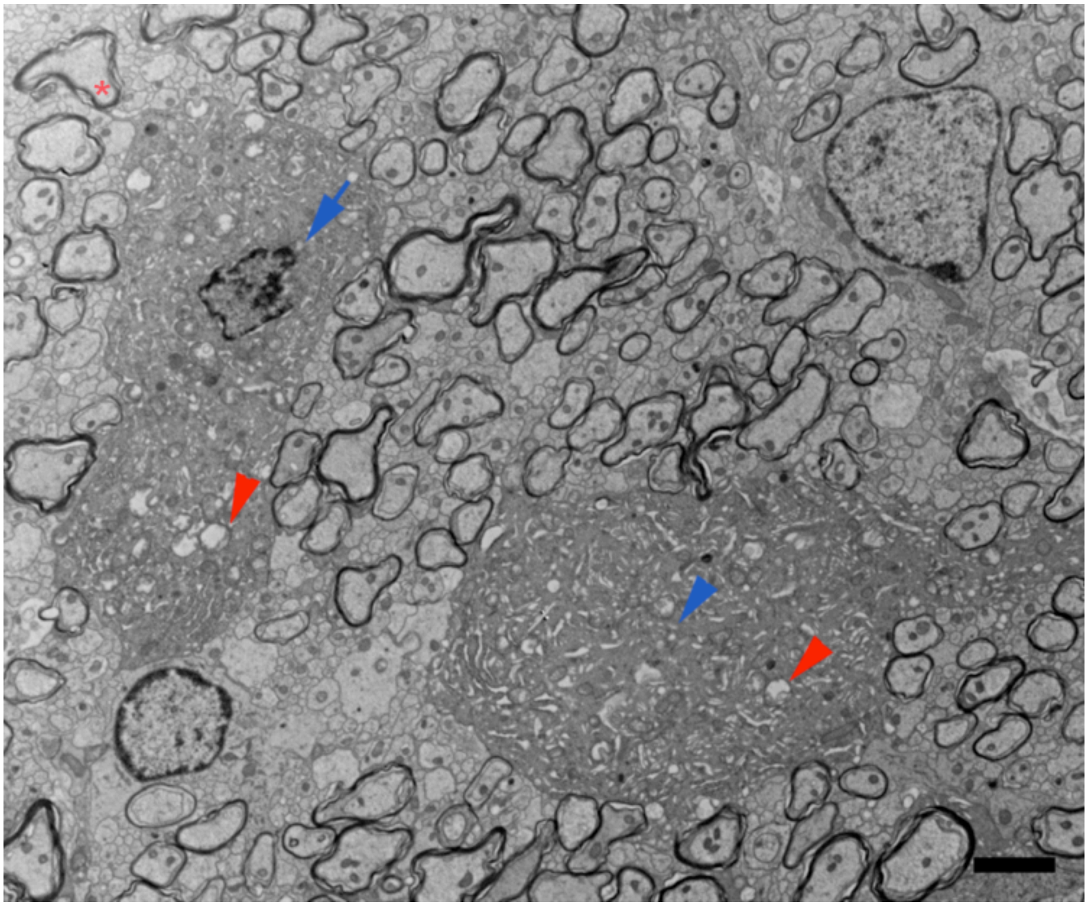

Supplementary Figure 4. Vacuole features in dying oligodendrocytes in the spinal cord of *Tsc1*-mutant mice

Representative EM image of dying OLs in *Tsc1*cKO mutant spinal cord at P14. Red arrowheads: vacuoles. Blue arrow and arrowhead: dying OLs with a shrunken nucleus and without a nucleus, respectively. Asterisk indicates a myelinating axon by a dying OL. Scale bar, 2 µm.

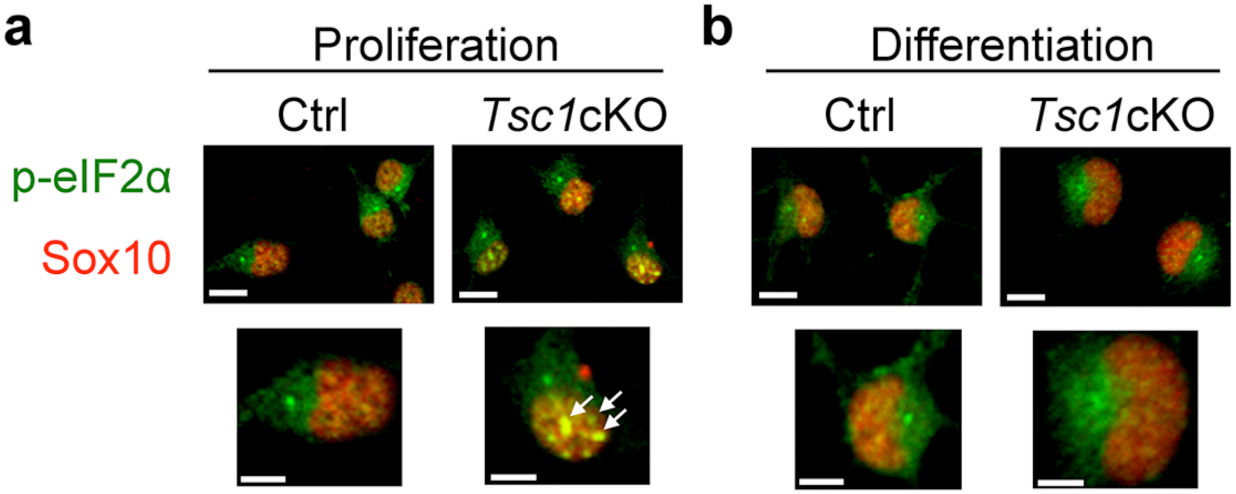

Supplementary Figure 5. Attenuation of p-eIF2α during the differentiation phase of *Tsc1*-mutant OPCs

(**a**,**b**) Upper panels: primary OPCs from control and *Tsc1*cKO cultured (**a**) under proliferation conditions or (**b**) in T3-containing differentiation medium for 24 h immunostained for p-eIF2α and Sox10, an OL lineage marker expressed in the nucleus. Scale bar, 10 μm.
Lower panels: a high magnification view of an OPC from control and *Tsc1*cKO under proliferation and differentiation conditions, respectively. Arrows indicate the p-eIF2α signal in the nucleus. Notably, the size of Sox10-labelled nuclei of mutant OPCs appeared larger in *Tsc1* mutants than controls under the differentiation condition. Scale bars, 3 μm.

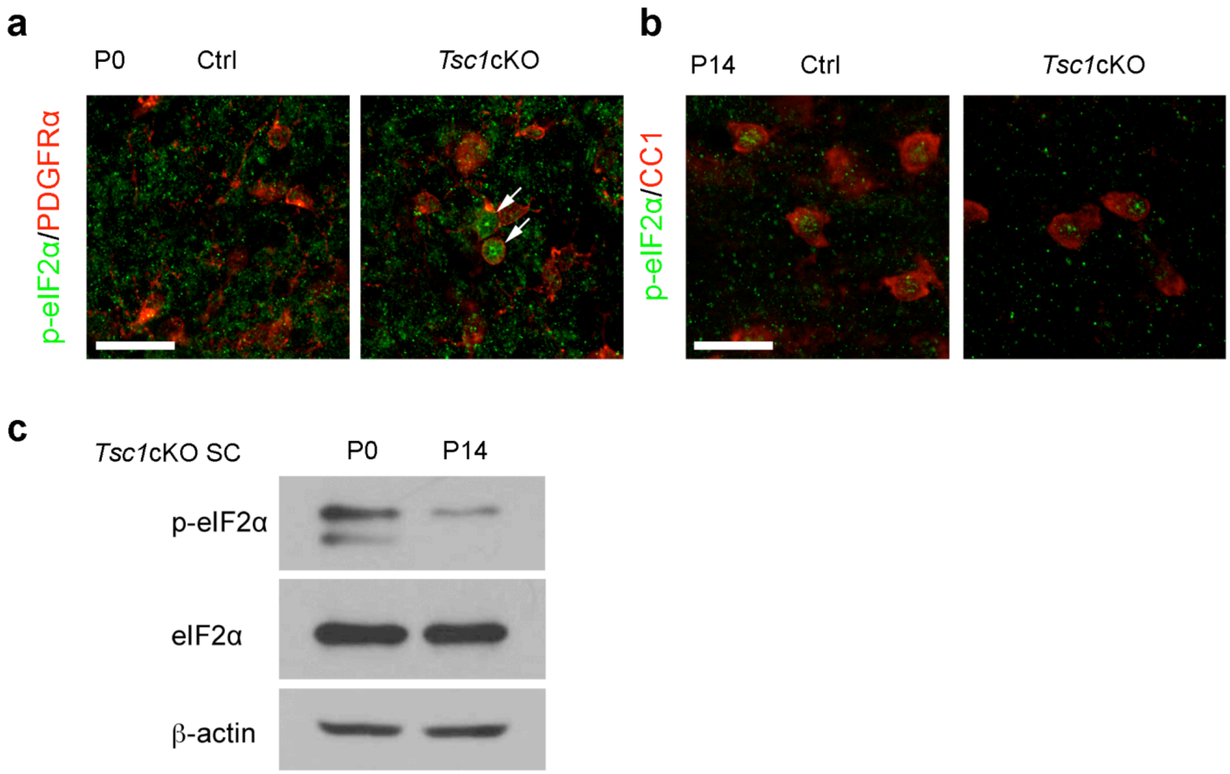

Supplementary Figure 6. Elevation of p-eIF2α in OPCs but not mature OLs in *Tsc1*cKO mice

(**a**) The corpus callosum of control and *Tsc1*cKO mice at P0 was immunostained with antibodies to PDGFRα and p-eIF2α. Scale bar, 25 μm.
(**b**) The corpus callosum of control and *Tsc1*cKO mice at P14 was immunostained with antibodies to CC1 and p-eIF2α. Scale bar, 25 μm.
(**c**) Representative western blot for p-eIF2α and eIF2α expression using spinal cord (SC) extracts of *Tsc1*cKO animals at P0 and P14. β-actin was used as a loading control.

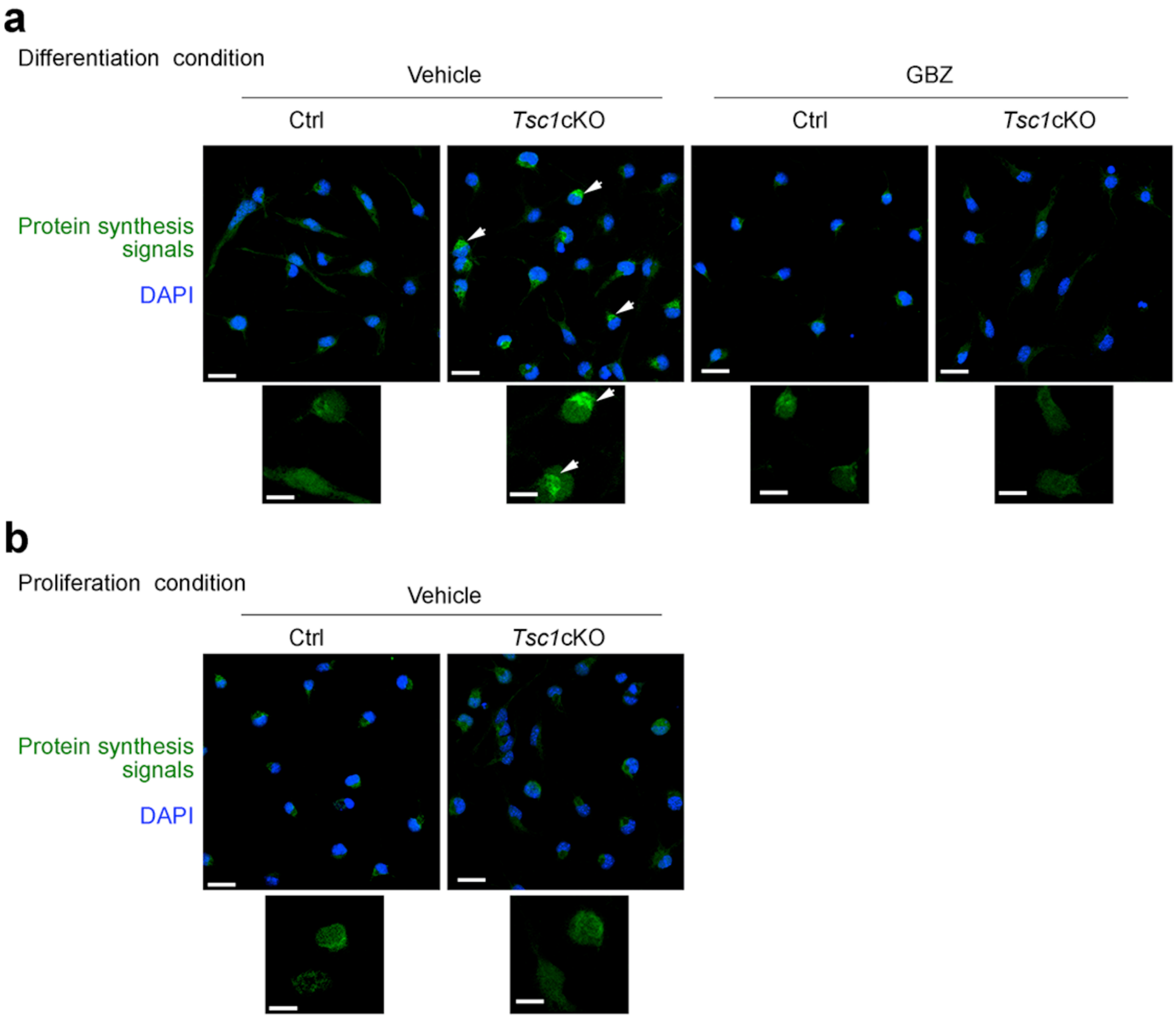

Supplementary Figure 7. Constitutive activating mTOR signaling elevates protein synthesis

(a,b) Upper panels: primary OPCs from control and *Tsc1*cKO were cultured under T3-containing differentiation medium for 24 h and treated with vehicle and guanabenz for 24 h (**a**) or under PDGFAA-containing proliferation medium with vehicle (**b**). Newly synthesized proteins were detected by Click-iT® HPG Alexa Fluor® 488 Protein Synthesis Assay Kit. Arrows indicate the signals of protein translation. Lower panels are shown at a high magnification. Scale bars in upper and lower panels in **a** and **b** are 10 and 3 μm, respectively.

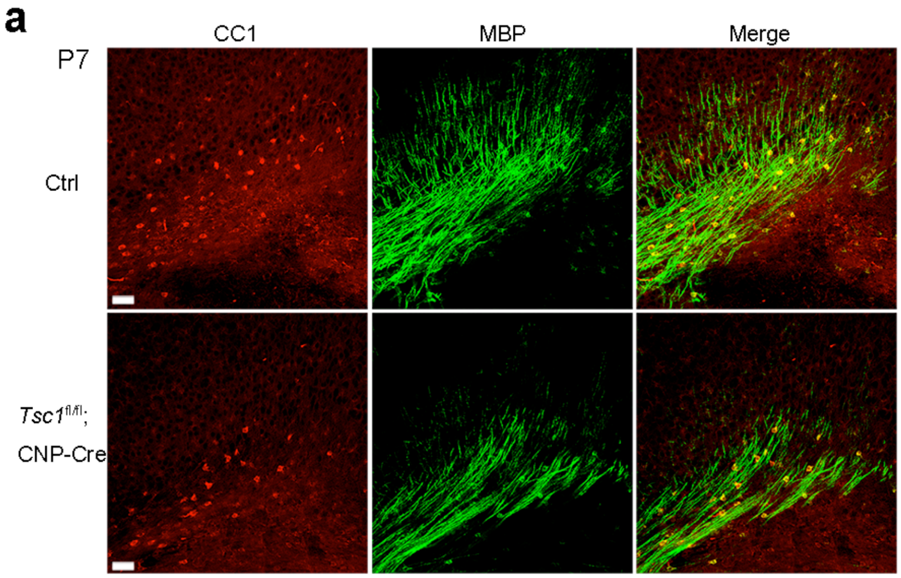

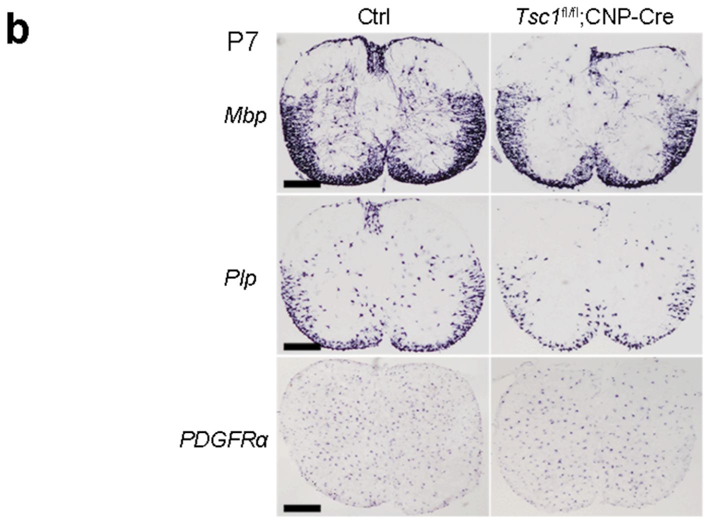

Supplementary Figure 8. Myelination deficiency in the CNS of *Tsc1* mutants in *CNP*-expressing cells

(**a**) The cortices of control and *Tsc1*$^{fl/fl}$;CNP-Cre$^{+/-}$ mutant were immunostained with antibodies to CC1 and MBP at P7. Scale bars, 50 µm.
(**b**) In situ hybridization to detect *MBP*, *Plp1*, and *PDGFRα* in spinal cords of control and *Tsc1*$^{fl/fl}$;CNP-Cre$^{+/-}$ mutant at P7. Scale bars, 100 µm

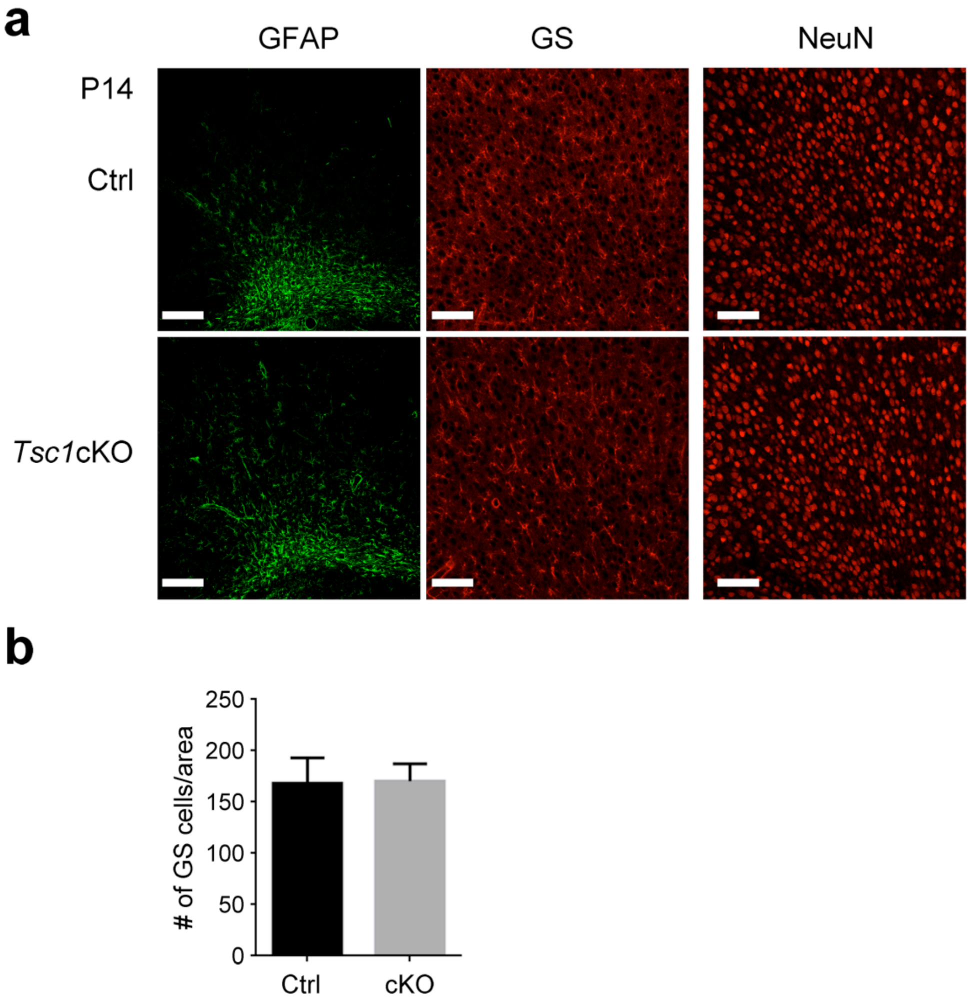

Supplementary Figure 9. Normal neuronal and astrocyte development in *Tsc1*cKO mice

(**a**) Images showing the cortices of control and *Tsc1*cKO mutants immunostained with antibodies to GFAP, GS and NeuN. Scale bars, 50 µm.

(b) Quantification of GS^+ cells in the cortex (0.4 mm^2) in control and *Tsc1*cKO mice at P14. Data represent the mean ± s.e.m. from three animals.

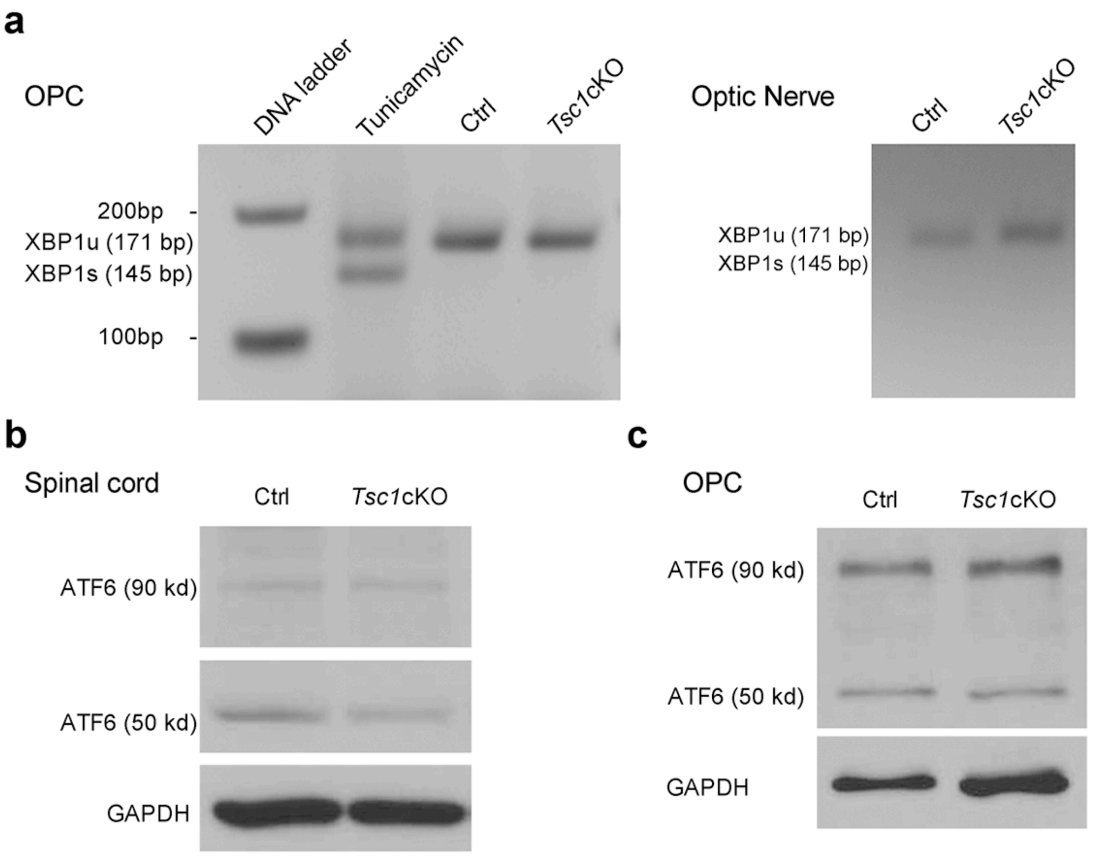

Supplementary Figure 10. *Tsc1* ablation induces ER stress independent of XBP-1 and ATF6

(**a**) PCR analysis of spliced forms of *XBP-1* in OPCs (left panel) under differentiation conditions or optic nerves (right panel) from controls and *Tsc1* mutants at P12. Tunicamycin (2 µg/ml) treated control OPCs were used as positive control.
(**b**) Western blot for ATF6 using spinal cords of control and *Tsc1*cKO animals at P14. GAPDH was used as a loading control.
(**c**) Western blot for ATF6 expression in OPCs isolated from control and *Tsc1*cKO mutants at P5. GAPDH was used as a loading control.

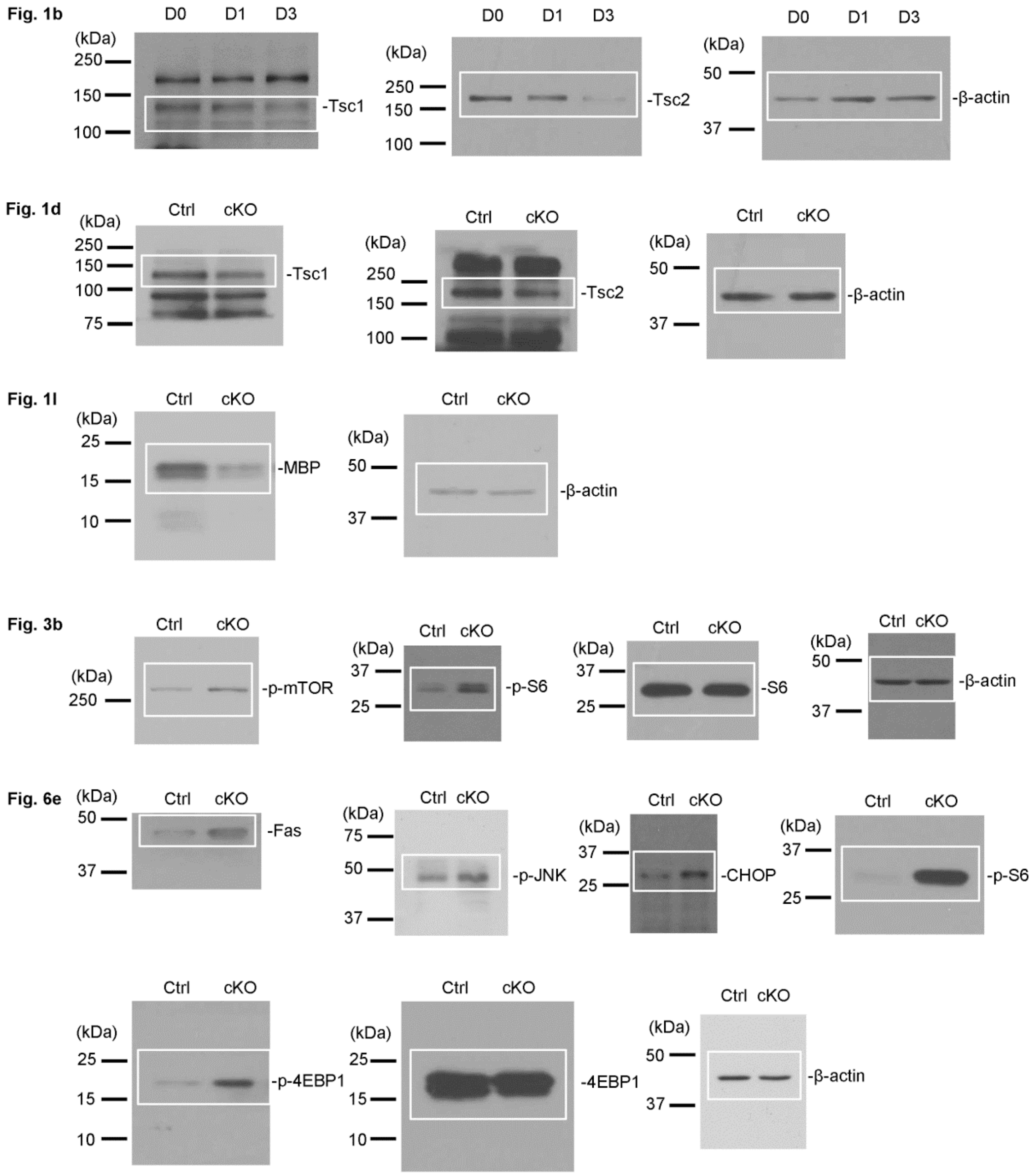

Supplementary Figure 11. Full-scanned images of western blots in the main Figures

Fig. 1b showing the western blots of Tsc1, Tsc2 and β-actin in rat OPCs after T3-treatment for 0, 1 and 3 days.

Fig. 1d showing the western blots of Tsc1, Tsc2 and β-actin in control and *Tsc1*cKO mutants.

Fig. 1i showing the western blots of p-mTOR, p-S6, S6 and β-actin in control and *Tsc1*cKO mutants.

Fig. 6e showing the western blots of Fas, p-JNK, CHOP, p-S6, p-4EBP1, 4EBP1 and β-actin.

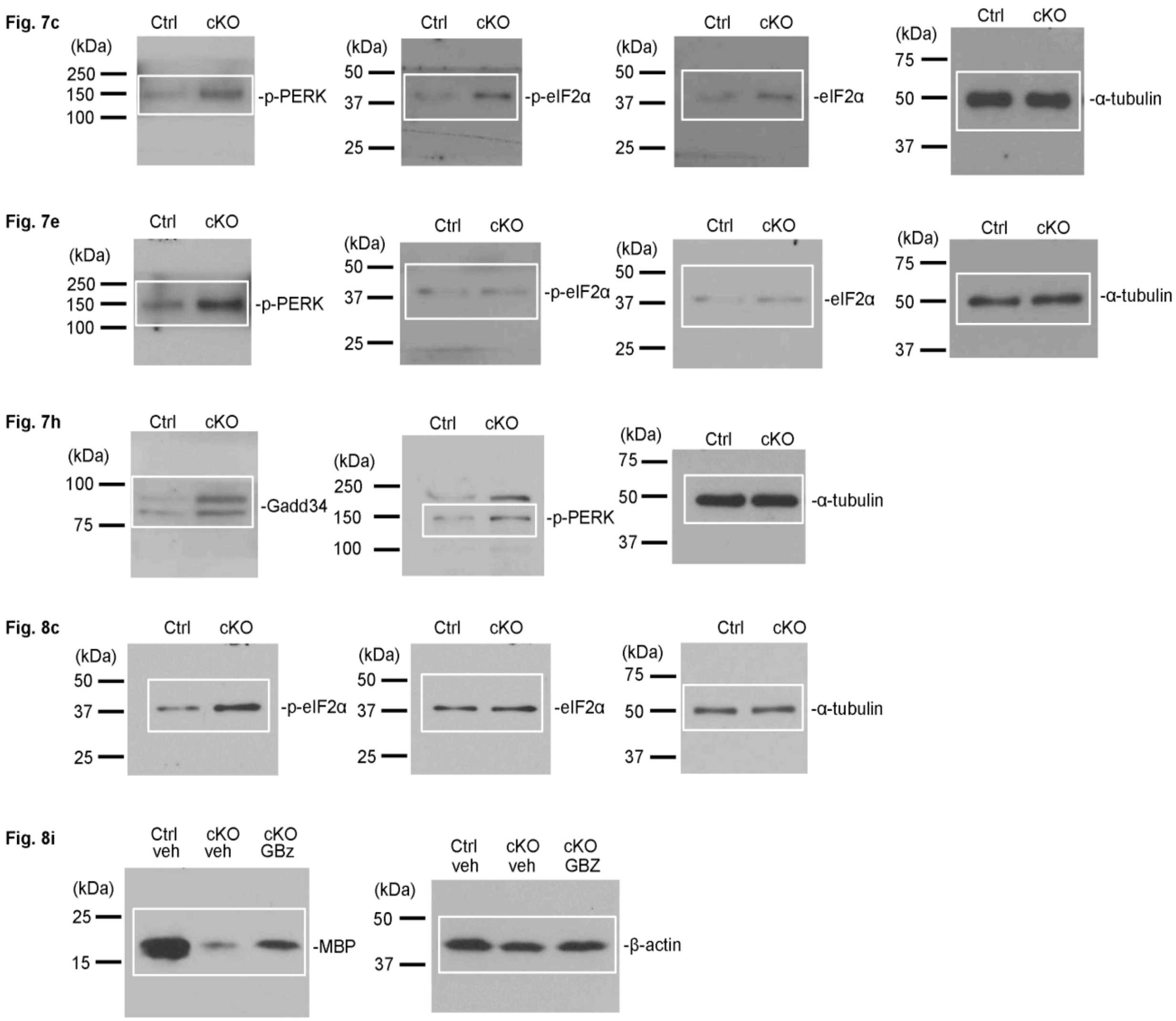

Supplementary Figure 12. Full-scanned images of western blots in the main Figures

Fig. 7c showing the western blots of p-PERK, p-eIF2α, eIF2α and α-tubulin blot under PDGFAA containing proliferation medium.

Fig. 7e showing the western blots of p-PERK, p-eIF2α, eIF2α and α-tubulin blot under T3-containing differentiation medium.

Fig. 7h showing the western blots of Gadd34, p-PERK and α-tubulin.

Fig. 8c showing the western blots of p-eIF2α, eIF2α and α-tubulin.

Fig. 8i showing the western blots of MBP and β-actin in control and *Tsc1*cKO mice treated with vehicle or guanabenz.

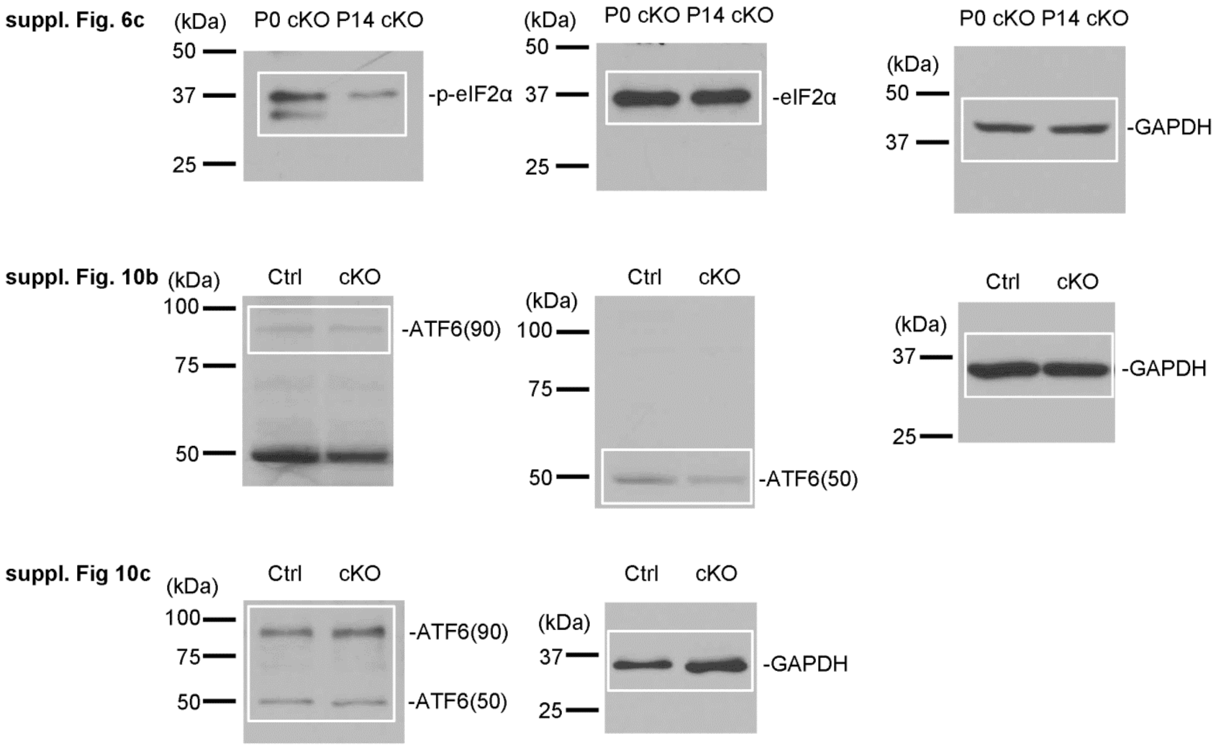

Supplementary Figure 13. Full-scanned images of western blots in the Supplementary Figures

Suppl. Fig. 6c showing the western blots of p-eIF2α, eIF2α and GAPDH.

Suppl. Fig. 10b showing the western blots of ATF6 and GAPDH.

Suppl. Fig. 10c showing the western blots of ATF6 and GAPDH.
